# Common variable immunodeficiency disorder-related liver disease is common and results in portal hypertension and an increased risk of death

**DOI:** 10.1097/HC9.0000000000000322

**Published:** 2023-12-15

**Authors:** Neil Halliday, Nadia Eden, Henry Somers, Niall Burke, Hiroshi Silva, Camila GX Brito, Andrew Hall, Alberto Quaglia, Siobhan O. Burns, David M. Lowe, Douglas Thorburn

**Affiliations:** 1 UCL Institute for Liver and Digestive Health, University College London, London, UK; 2 Sheila Sherlock liver centre, Royal Free London NHS Foundation Trust, London, UK; 3 Department of Cellular Pathology, Royal Free London NHS Foundation Trust, London, UK; 4 Institute of Immunity and Transplantation, University College London, UK; 5 Department of Immunology, Royal Free London NHS Foundation Trust, London, UK

## Abstract

**Background:**

Common variable immunodeficiency disorder (CVID) manifests with recurrent infections and inflammatory complications, including liver disease. We report the clinical features, natural history, and outcomes of patients with CVID-related liver disease (CVID-rLD) from a tertiary immunology and hepatology center.

**Methods:**

Two hundred eighteen patients were identified; CVID-rLD was defined by persistently abnormal liver function tests or evidence of chronic liver disease (CLD) or portal hypertension (PHTN) by radiological or endoscopic investigation, after exclusion of other causes. Patients with CVID-rLD were investigated and managed following a joint pathway between immunology and hepatology services. Data, including clinical parameters, investigations, and outcomes, were retrospectively collected.

**Results:**

A total of 91/218 (42%) patients had evidence of CVID-rLD, and 40/91 (44%) had PHTN. Patients with CVID-rLD were more likely to have other noninfectious complications of CVID (85/91, 93.4% vs. 75/127, 59.1%, *p*<0.001) including interstitial lung disease, gut disease, and autoimmune cytopenias. Nodular regenerative hyperplasia (NRH) was identified in 63.8% of liver biopsies, and fibrosis in 95.3%. Liver stiffness measurements (LSMs) were frequently elevated (median 9.95 kPa), and elevated LSM was associated with PHTN. All-cause mortality was higher in those with CVID-rLD (24/91, 26.4% vs. 14/127, 11%, *p*=0.003), which was the only organ complication associated with mortality (HR 2.24, 1.06–4.74, *p*=0.04). Factors predicting mortality in CVID-rLD included PHTN, increasing fibrosis, and LSM.

**Conclusions:**

Liver disease is a common complication of CVID as part of complex, multi-organ involvement and is associated with high rates of PHTN and an increased hazard of mortality.

## Graphical abstract

**Figure:**
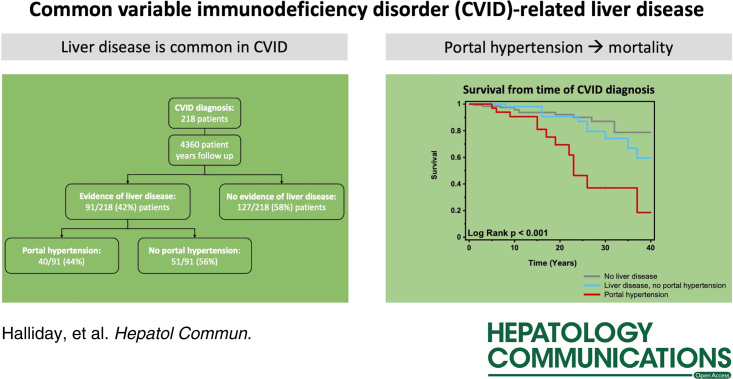

## INTRODUCTION

Common variable immunodeficiency disorder (CVID) is a clinically heterogenous condition characterized by hypogammaglobulinemia and poor vaccine responses and is the commonest primary immunodeficiency disorder in adults.^1^ CVID can manifest with an infection-predominant phenotype or with complex CVID, characterized by infection, immune dysregulation, and organ inflammation. Noninfectious complications include autoimmunity, cytopenias, granulomatous lymphocytic interstitial lung disease (GLILD), lymphoid hyperplasia, disease, an elevated risk of malignancy, and liver disease.^2,3^

Varied patterns of liver disease are observed in CVID; historically, viral hepatitis was a leading cause,^4^ although with blood product screening and effective treatments this is now much diminished. Other patterns of liver disease include sclerosing cholangitis, primary biliary cholangitis or autoimmune hepatitis-like conditions, granulomatous hepatitis, and nodular regenerative hyperplasia-like changes (NRH-LCs).^5,6^ A proportion of patients progress to cirrhosis and end-stage liver disease.^6,7^ The commonest liver pathology observed is nodular regenerative hyperplasia (NRH), with or without an inflammatory infiltrate or granulomatous hepatitis, often with characteristic pericellular fibrosis.^5,6^ Sequelae of NRH include noncirrhotic portal hypertension (PHTN) and its complications.^5,7^

Prevalence estimates for nonviral liver disease in CVID (hereby termed CVID-related liver disease (CVID-rLD) range from 5% to 79%,^2–4,7–12^ reflecting the differing cohorts studied, definitions of liver disease, and approaches to screening. However, CVID-rLD has been associated with adverse outcomes, with an elevated hazard of mortality and accelerated time to death.^7,13^ It remains unclear whether CVID-rLD drives mortality or rather identifies a more severe CVID phenotype. Additionally, the risk factors and natural history of CVID-rLD have not been well defined.

As NRH can lead to clinically significant PHTN, the early detection and risk stratification of patients with CVID are an important unmet need. Current recommendations suggest the use of noninvasive tests, particularly liver stiffness measurements (LSM), to identify those at risk of clinically significant PHTN in cirrhosis^14^; however, these strategies are not applicable in portosinusoidal vascular disorders, including NRH-LCs in CVID, as the pattern and degree of fibrosis differ from cirrhotic liver disease.^6^ However, changes in LSM have been shown to be associated with liver enzyme abnormalities, histological changes of NRH, and the presence of complications, including PHTN and enteropathy in CVID.^9,10,15^ We have, therefore, explored the use of LSM in the clinical context of CVID-rLD for the prediction of endoscopic or radiological evidence of PHTN.

Here we report the outcomes for a large cohort of patients with CVID followed up at a single tertiary immunology and hepatology center and demonstrate the prevalence, natural history, and clinical outcomes for patients with CVID-rLD. We describe the clinical features associated with CVID-rLD, the risk factors for mortality, and the clinical utility of diagnostic tests for the identification of PHTN.

## METHODS

### Patient cohort

All patients with a diagnosis of CVID, who had undergone at least 2 clinical reviews at our center, were identified from our patient database, which was constructed in 2000 with the retrospective inclusion of historical patients and prospective inclusion of new patients thereafter. CVID was diagnosed by a consultant immunologist in line with internationally accepted guidelines^1^ with a typical presentation including infection, hypogammaglobulinemia, and failure to respond to immunization.

All patients were treated with i.v. or subcutaneous immunoglobulin, targeting a trough IgG level of >8 g/L. Clinical management was personalized for each patient but characterized by prompt treatment of infection, prophylactic antibiotics where indicated, and typically 6 monthly monitoring of liver function tests (LFTs). GI endoscopy and microbiological investigations were undertaken as clinically indicated, CT scanning of the thorax ~5-yearly and pulmonary function tests every 1–2 years, unless more frequent testing was indicated. All research was conducted in accordance with both the Declarations of Helsinki and Istanbul.

### Liver evaluation

Prior to 2010, patients were referred to hepatology services based on clinical need. From 2010 onward, a formalized pathway for hepatology review was established; all patients underwent a baseline abdominal ultrasound and LFT monitoring. Those with abnormal LFTs, evidence of PHTN, or chronic liver disease (CLD) were reviewed in hepatology clinics or from 2016 in a joint clinic with both an immunologist and a hepatologist.

Full hepatological assessment with a liver disease etiological screen was undertaken in the liver clinic, including estimation of liver stiffness by transient elastography using Fibroscan wherever possible. Etiological screen included screening for hepatotropic viruses by nucleic acid tests (as serology is not representative in these patients) including for hepatitis B and C and more recently for hepatitis E, screening for autoantibodies ( antinuclear, antismooth muscle, antiliver, and kidney microsomal), ferritin and transferrin saturation, caeruloplasmin, and alpha 1 antitrypsin. A liver biopsy was undertaken when investigations were suspicious for CLD. All liver biopsies were re-reviewed for this study and assessed for the presence of granulomata, NRH, fibrosis, steatosis, and features of cholangiopathy. Fibrosis stage was scored using a dedicated 4-tier scoring system following picro-Sirius red staining: stage 0: no fibrosis; 1: patchy and focal pericellular fibrosis; 2: diffuse pericellular fibrosis; 3: bridging fibrosis; 4: bridging fibrous septa encasing parenchymal nodules/cirrhosis. In view of the coexistence of NRH and fibrosis in CVID, we have adopted the term NRH-LC as proposed by Crotty et al^6^ to refer to typical changes of NRH accompanied by inflammatory infiltrate or fibrosis. When biopsies were performed by means of the transjugular route, HVPG was measured by standard techniques.^16^

### Definitions of organ involvement

Liver disease was defined by the presence of persistently abnormal serum transaminases or alkaline phosphatase on 2 consecutive occasions at least 6 weeks apart or by radiological evidence of CLD or evidence of PHTN. Evidence for PHTN included radiographic stigmata (portosystemic vascular shunts, ascites, reversal of hepatopetal portal vein flow or recanalization of the umbilical vein), endoscopic stigmata (esophageal, gastric or ectopic varices, or PHTN gastropathy), or HVPG ≥ 6 mm Hg. Isolated splenomegaly was not accepted as indicating PHTN.

GI involvement was defined as chronic diarrhea, that is, patients experiencing loose bowel motions >3 times per day for at least 6 weeks. GI infections were identified by microscopy and culture or fecal nucleic acid tests for pathogenic viruses (eg, norovirus), *Giardia lamblia*, *Campylobacter spp.*, *Salmonella spp.*, *Shigella spp.*, and pathogenic *Escherichia coli*, or on duodenal histology or duodenal aspirates for *G. lamblia*. Inflammatory bowel disease was diagnosed on typical endoscopic and histological findings. Patients with chronic diarrhea in the absence of infection or inflammatory bowel disease were labeled as having “enteropathy.”

Immune-mediated cytopenias were defined on clinical grounds. Interstitial lung disease (ILD) was based on high-resolution CT of the lungs and pulmonary function tests, and bronchiectasis on CT appearances. Splenomegaly was defined as the longitudinal dimension of the spleen >12 cm on imaging.

### Patient exclusions

Patients with other causes for liver disease, including suspected alcohol-associated liver disease, alpha 1 antitrypsin deficiency, and chronic viral hepatitis, were excluded (7 patients).

### Ethics

Ethical approval was not required as the clinical data collected were as part of routine clinical care pseudonymized and analyzed by the clinical care team. Retrospective histological review of liver biopsy specimens was undertaken under ethical approval Ref: 07/Q0501/50 granted by the Hampstead REC.

### Statistical methods

Student’s *t* tests, χ^2^ tests, and log-rank tests were used to test continuous, categorical, and failure rate variables as appropriate. OR was calculated from binary logistic regression analyses. Time to event modeling by Cox-proportionate hazard models was undertaken with clinically relevant variables for univariable analysis (sex, age, era, autoimmune complications, other organ system involvement of CVID, and exposure to immunosuppressive treatments). Multivariable models were constructed utilizing forward selection of variables where *p* ≤ 0.1 in univariable analysis. Cutoff thresholds for diagnostic tests were determined by the Youden index from sensitivity and specificity data for the test in question. Statistical significance was taken at *p*<0.05. Cases with missing data were excluded as stated. All analyses were performed with SPSS Statistics Version 27 (IBM) and OriginPro2020b (OriginLab).

## RESULTS

### Characteristics of the patient cohort

Two hundred eighteen patients with CVID were identified and followed up for a median of 17 years (IQR 11–26). Liver disease occurred in 91/218 patients (42%). The median age at CVID diagnosis was 32 years, and 100 patients (45.7%) were male. Clinical characteristics are shown in Table 1. There were no differences in sex, age at diagnosis of CVID, decade of diagnosis, or the duration of follow-up between those with and without liver disease (Table 1 and Supplemental Figures S1 and S2, http://links.lww.com/HC9/A670).

**TABLE 1:** Clinical and demographic features of CVID patient cohort

	Liver disease n=91 (44 for small bowel histology assessment)		No liver disease n=127 (116 for lung, 115 for spleen, and 40 for small bowel histology assessments)		Total		*p*
Sex							
Male	41	45.1	59	46.5	100	45.9	0.84
Age at CVID diagnosis (y)	32	(21–41)	33	(20–49)	32	(20–45)	0.34
Decade of diagnosis							
‘60–’79	9	9.9	13	10.2	22	10.1	0.73
‘80–’99	31	34.8	50	39.4	81	37.2	—
‘00–	51	56.0	64	50.4	115	52.7	—
Follow-up (y)	17	(13–27)	18	(11–26)	17	(11–26)	0.55
Lung disease n=116 no LD							
Any	**71**	**78.0**	**69**	**59.5**	**140**	**67.6**	**0.005**
ILD/GLILD	**33**	**36.3**	**13**	**11.2**	**46**	**22.2**	**<0.001**
Bronchiectasis	61	67.0	65	56.0	126	61.2	0.12
Splenomegaly n=115 no LD							
>12 cm	**65**	**71.4**	**43**	**37.4**	**109**	**52.9**	**<0.001**
Splenectomy	**7**	**7.7**	**3**	**1.4**	**10**	**4.6**	**0.02**
GI disease							
Any	**60**	**67.0**	**57**	**44.9**	**117**	**53.7**	**0.002**
Chronic diarrhea	**52**	**57.1**	**47**	**37.0**	**99**	**45.4**	**0.003**
“Enteropathy”	**49**	**53.8**	**37**	**29.1**	**86**	**39.4**	**<0.001**
IBD-like	8	8.8	7	5.5	15	6.9	0.35
Villous blunting	11	25	10	25.0	22	23.9	1
Infective	21	23.1	26	20.5	47	21.6	0.64
Norovirus	8	8.8	4	3.1	12	5.5	0.07
Giardia	11	12.1	12	9.4	23	10.6	0.53
Other	6	6.6	12	9.4	18	8.3	0.45
Joint disease							
Arthritis	12	13.2	11	8.7	23	10.6	0.28
Cytopenia							
Any	**34**	**37.4**	**22**	**17.3**	**56**	**25.7**	**<0.001**
ITP	**33**	**36.3**	**17**	**13.4**	**50**	**22.9**	**<0.001**
Autoimmune hemolytic anemia	11	12.1	8	6.3	19	8.7	0.14
Autoimmune neutropenia	7	7.7	4	3.1	11	5.0	0.13
Other autoimmunity	14	15.4	22	17.3	36	16.5	0.70
Any immune-mediated complication	**85**	**93.4**	**75**	**59.1**	**160**	**73.4**	**<0.001**

All bold values have a *p* value in the table.

*Note:* Data presented as n and % or (IQR).

Abbreviations: CVID, common variable immunodeficiency disorder; GLILD, granulomatous lymphocytic interstitial lung disease; IBD, inflammatory bowel disease; ILD, interstitial lung disease; LD, liver disease.

All 91 patients with liver disease and 116/127 (91.3%) without underwent CT chest imaging. Abdominal imaging with ultrasonography, CT, or MRI was performed in 90/91 (98.9%) of those with liver disease and 115/127 (90.6%) of those without (see Supplemental Table S1, http://links.lww.com/HC9/A670 for investigation details).

### Clinical associations with CVID-rLD

Liver disease was associated with the presence of other inflammatory conditions and autoimmune cytopenias (Table 1). Among those with liver disease, ILD was more prevalent (33/91, 36.3% vs. 13/127, 11.2%, *p*<0.001), as were splenomegaly (65/91, 71.4% vs. 43/127, 37.4%, *p*<0.001), enteropathy (49/91, 53.8% vs. 37/127, 29.1%, *p*<0.001), cytopenias (34/91, 37.4% vs. 22/127, 17.3%, *p*<0.001), and specifically idiopathic thrombocytopenic purpura (33/91, 36.3% vs. 17/127, 13.4%, *p*<0.001) (Table 1). Overall, 85 (93.4%) of the patients with liver disease had at least 1 immune-mediated comorbidity compared to 75 (59.1%) without liver disease (*p*<0.001).

Of 91 patients with liver disease, 49 (53.8%) experienced immunosuppressive medication, similar to those without liver disease [57/127 (44.9%)], with corticosteroids the commonest agent used (Supplemental Table S2, http://links.lww.com/HC9/A670). Mycophenolate and rituximab were used more frequently in those with liver disease (13.2% vs. 3.9%, *p*=0.012 and 16.5% vs. 3.9%, *p*=0.002, respectively).

We next tested which comorbidities were associated with a greater odds of liver disease. Liver disease was more common in patients with granulomatous lymphocytic ILD (OR 2.82, 95% CI: 1.23–6.47, *p*=0.014), splenomegaly (including splenectomy) (OR 5.82, 95% CI: 2.84–11.88, *p*<0.001), and chronic diarrhea (OR 2.61, 95% CI: 1.38–4.95, *p*=0.003) in adjusted binary logistic regression analysis (Table 2).

**TABLE 2:** Association of liver disease with other complications of CVID

		Univariate				Multivariate			
	Liver disease		95% CI				95% CI		
	n (%)	OR	Lower	Upper	*p*	OR	Lower	Upper	*p*
Granulomatous lymphocytic interstitial lung disease (n=46)	**33** (**71.7)**	**5.00**	**2.44**	**10.20**	**<0.001**	**2.82**	**1.23**	**6.47**	**0.014**
Splenomegaly/splenectomy (n=119)	**73** (**61.3)**	**7.14**	**3.80**	**13.41**	**<0.001**	**5.80**	**2.84**	**11.88**	**<0.001**
Lymphadenopathy (n=52)	**30** (**57.7)**	**2.35**	**1.24**	**4.43**	**0.008**	1.08	0.51	2.29	0.834
Arthritis (n=22)	11 (50)	1.45	0.60	3.51	0.409	—	—	—	—
Chronic diarrhea (n=99)	**52** (**52.5)**	**2.27**	**1.31**	**3.93**	**0.003**	**2.61**	**1.38**	**4.95**	**0.003**
Autoimmune cytopenia (n=55)	**34** (**61.8)**	**2.72**	**1.45**	**5.09**	**0.002**	0.99	0.44	2.22	0.984

Note: All bold values have a *p* value in the table.

Abbreviation: CVID, common variable immunodeficiency disorder.

**FIGURE 1:**
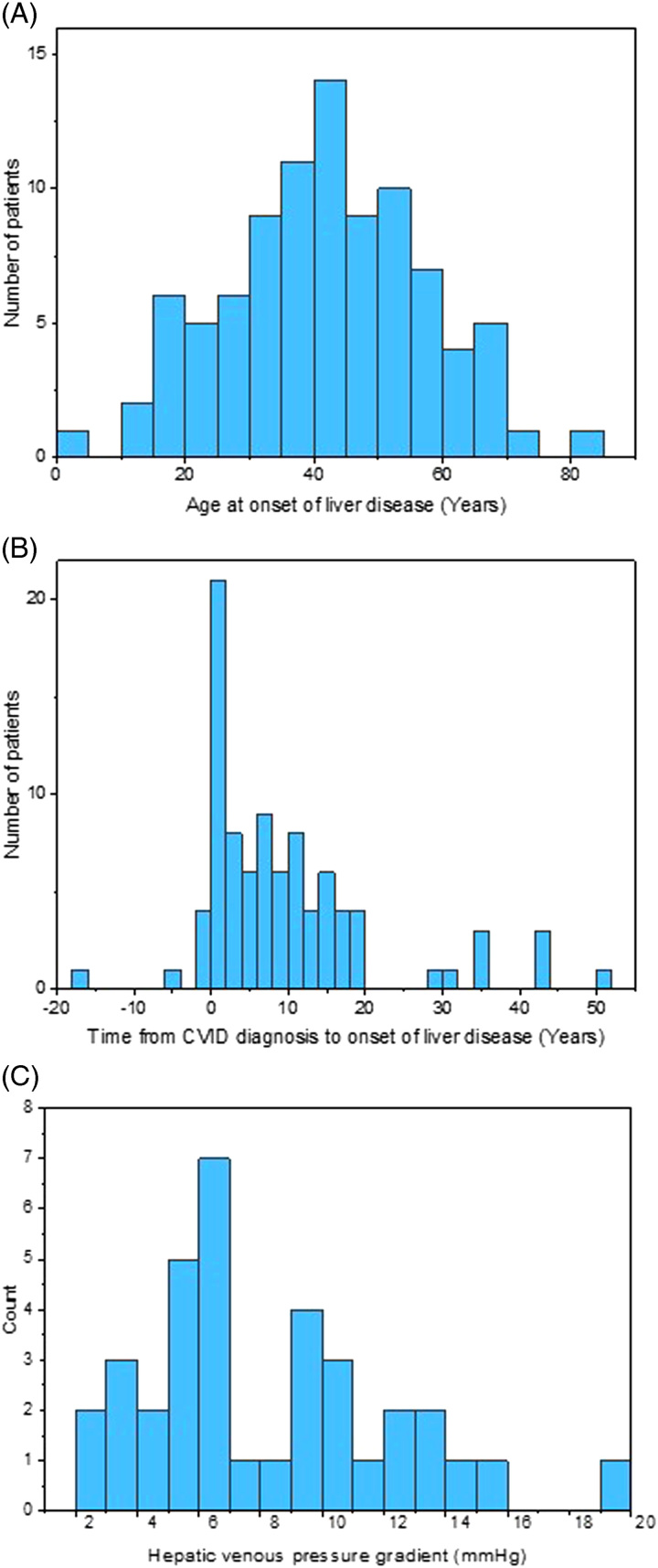
(A) Histogram of the age of onset of liver disease among patients with CVID. (B) Histogram of time from CVID diagnosis to first evidence of liver disease. (C) Histogram of Hepatic venous pressure gradient measurements of patients with CVID-related liver disease. Abbreviation: CVID, common variable immunodeficiency disorder.

The median age of liver disease onset (defined as the first abnormal LFTs, or evidence of liver disease or PHTN) was 41 years (IQR 31–53) (Figure 1A). The onset of liver disease was generally an early event following the diagnosis of CVID, with median time to liver disease of 6 years (IQR 1–14), although liver disease predated CVID diagnosis in some (Figure 1B).

### Histological features of CVID-rLD

Among patients with liver disease, 47 (51.7%) had a liver biopsy suitable for review (Supplemental Table S3, http://links.lww.com/HC9/A670). Liver biopsy was taken a median of 13 years after the diagnosis of CVID (IQR 7–19) and a median of 6 years after the first abnormal liver enzyme measurement (IQR 2–9). NRH-LC was a common finding, evident in 30/47 (63.8%) of biopsies. Granuloma was present in 11/47 (23.4%); in 10/11 of those with granulomas, NRH-LC was also present. Fibrosis was commonly observed, typically in a pericellular pattern (stages 1–2) with the additional formation of bridging fibrous septa, with or without the formation of parenchymal nodules (stages 3–4). In the 43 biopsies where assessment of fibrosis was possible, stage 3 or 4 fibrosis was observed in 22 (51.1%). The majority of biopsies (38/47, 80.9%) demonstrated an inflammatory, lymphocytic infiltrate, typically of mild to moderate severity, in the periportal and/or lobular regions, with intrasinusoidal lymphocytes frequently observed.

### Liver stiffness and PHTN in CVID-rLD

LSM by Fibroscan was performed in 40/91 (44%) patients at a median of 13.5 years following the diagnosis of CVID (IQR 9-23) and 8 years after first abnormal liver enzymes (IQR 4.5–12). A median LSM of 9.95 kPa (IQR 6.8–14.55) was observed with evidence of advanced fibrosis with an LSM >13.5 kPa seen in 10/40 (25%) patients (Table 3).

**TABLE 3:** Results of Fibroscan, endoscopic, radiological, and HVPG measurements of patients with CVID-related liver disease

Liver disease investigations	N/median	IQR (%)
Transient elastography n=40		
Median LSM (kPa)	9.95	6.8–14.55
LSM >13.5 kPa	10	(25)
Endoscopic evidence of portal hypertension n=62	21	(33.9)
Radiological evidence of PHTN n=90	24	(26.7)
HVPG n=36		
Median HVPG	6	5–10
HVPG ≥6	24	(67.6)
HVPG ≥10	11	(29.7)

Abbreviations: CLD, chronic liver disease; CVID, common variable immunodeficiency disorder; LSM, liver stiffness measurement; PHTN, portal hypertension.

Endoscopy was performed in 62/91 (68%) patients at a median of 13 years after CVID onset (IQR 6–20) and 4 years after first observed abnormal liver enzymes (IQR 2–8). Evidence of PHTN was observed at endoscopy in 21/62 (33.9%) (2 patients with PHTN gastropathy without varices had evidence of portosystemic shunts on abdominal imaging).

Abdominal imaging was performed in 90/91 (99%) of patients, a median of 9 years after CVID diagnosis (IQR 2-16) and 1 year after first abnormal liver enzymes (IQR 0–5). Stigmata of PHTN were seen on abdominal imaging in 24/90 (26.7%).

HVPG measurement was undertaken in 36 patients at 13 years (IQR 7–18.5) after CVID diagnosis and 6 years (IQR 3–9) after onset of abnormal liver enzymes. The median HVPG was 6 mm Hg (range 2–19 mm Hg) (Table 3, Figure 1C).

PHTN, defined as any of endoscopic or radiographic evidence or HVPG ≥6 mm Hg, was observed in 44.0% of patients with liver disease, with an HVPG threshold of ≥10 mm Hg PHTN was observed in 37.4% (Table 4). The median time to onset of PHTN was 10–10.5 years after the diagnosis of CVID and 4.5–5 years after the first evidence of liver disease (Table 4).

**TABLE 4:** Clinical outcomes of death, decompensated liver disease, and evolution of PHTN in patients with CVID-related liver disease.

	Liver disease					
		Time to event, years (IQR)		No liver disease		
Clinical outcomes	n (%)	From CVID diagnosis	From liver disease diagnosis	n (%)	Time from CVID diagnosis, years (IQR)	*p*
Death	**24** (**26.4)**	**22.5** (**5–51)**	**10** (**0–35)**	**14** (**11)**	**15** (**1–55)**	**0.003**
Decompensated CLD	10 (11.0)	11.5 (3–26)	5.5 (0–11)	—	—	—
PHTN (radiological/endoscopic/HVPG HVPG ≥6 mm Hg)	40 (44.0)	10.5 (0–48)	4.5 (0–15)	—	—	—
PHTN (radiological/endoscopic/HVPG ≥10 mm Hg)	34 (37.4)	10 (0–48)	5 (0–15)	—	—	—

Note: All bold values have a *p* value in the table.

Abbreviations: CLD, chronic liver disease; CVID, common variable immunodeficiency disorder; range; PHTN, portal hypertension.

There were no clinical differences between patients with liver disease who had LSM or not, other than those who did were more likely to have PHTN (Supplemental Table S4, http://links.lww.com/HC9/A670). Clinical characteristics were similar between those who did and did not undergo endoscopy, other than the presence of splenomegaly and GI involvement being more common in those who underwent endoscopy (Supplemental Table S5, http://links.lww.com/HC9/A670). Likewise, the characteristics of those who underwent HVPG measurement or liver biopsy were broadly similar to those who did not, although they were more likely to have respiratory involvement, villous blunting on small bowel biopsy, and splenomegaly (Supplemental Tables S6 and S7, http://links.lww.com/HC9/A670).

### Associations with clinical PHTN

We next tested the associations between clinical assessments of PHTN in patients with CVID-rLD. As NRH leads to presinusoidal PHTN with only modest elevations in HVPG, as expected, mean HVPG was not significantly higher in those with evidence of PHTN (mean HVPG 7.1 vs. 8.1 mm Hg, *p*=0.48) (Supplemental Figure S3A, http://links.lww.com/HC9/A670). A total of 41.7% (5/12) of patients with an HVPG ≤ 5 mm Hg had clinical evidence of PHTN. Consequently, HVPG was a poor predictor of PHTN [AUROC 0.574, *p*=0.447 (Supplemental Figure S3B, http://links.lww.com/HC9/A670)]. However, the presence of NRH-LC on biopsy was significantly associated with PHTN as assessed by endoscopy, imaging, or HVPG ≥6 mm Hg (*p*=0.002) or ≥10 mm Hg (*p*=0.02).

**FIGURE 2:**
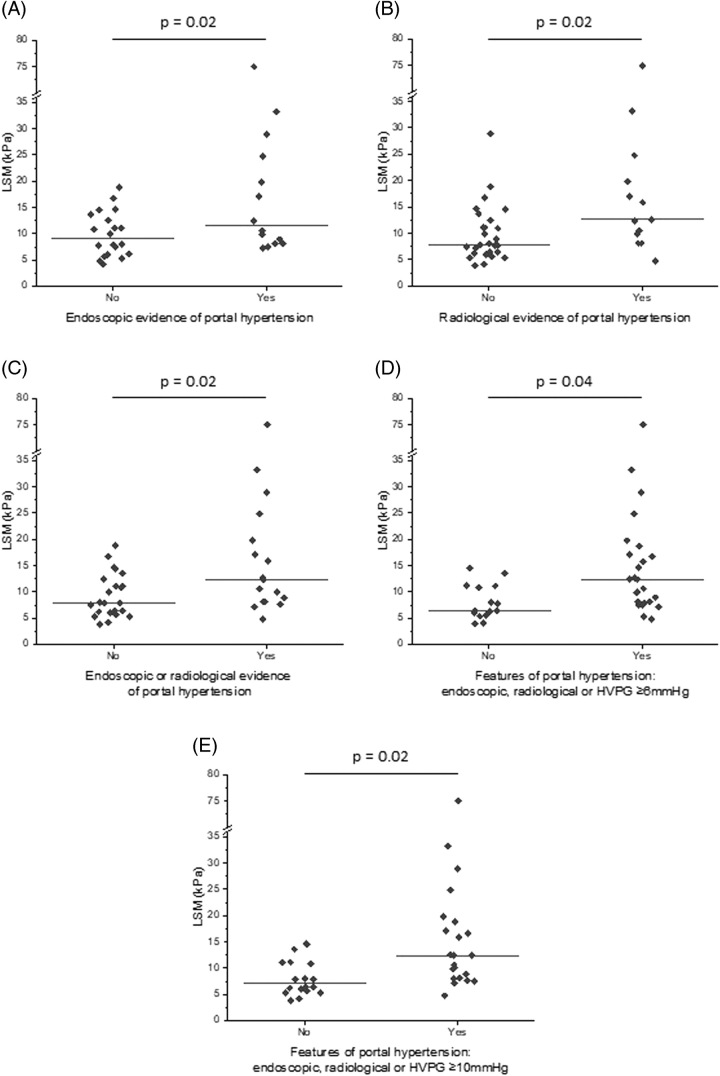
Liver stiffness measurement observed for different clinical measures of PHTN endoscopy (A), radiology (B), endoscopy of radiology (C), endoscopy or radiology or HVPG ≥ 6 mm Hg (D), and endoscopy or radiology or HVPG ≥ 10 mm Hg (E) for patients. *p*-values represent the Student *t* test. Abbreviation: LSM, liver stiffness measurement; PHTN, portal hypertension.

We next compared LSM with the presence of PHTN defined by endoscopic, radiological, or composite assessments (Figure 2). LSM was higher in those with endoscopic stigmata of PHTN [median 9 kPa (IQR 6.1–13.1) vs. 11.5 kPa (IQR 8.1–24.8), *p*=0.02], radiological stigmata [7.9 (6.2–12.4) vs. 12.6 (9.9–19.8) kPa, *p*=0.02], either endoscopic or radiological stigmata [7.9 (6–12.4) vs. 12.4 (8.1–19.8) kPa, *p*=0.02] endoscopic, radiological, or HVPG ≥6 mm Hg [6.4 (5.6–11.1) vs. 12.4 (8.1–17.1) kPa, *p*=0.04], or endoscopic, radiological, or HVPG ≥10 mm Hg [7.1 ( 5.6–11.1) vs. 12.4 (8.1–18.8) kPa, *p*=0.02] (Figure 2A–E).

### Mortality and end-stage liver disease in CVID-rLD

Adverse clinical outcomes were more common in patients with liver disease; crude mortality was greater in those with liver disease [24 (26.4%) patients] compared to those without [14 patients (11%) (*p*=0.003)]. Decompensated CLD (ascites, variceal hemorrhage, or spontaneous bacterial peritonitis) occurred in 10/91 (11.0%) of those with liver disease (Table 4).

Cox-proportional hazard models for death revealed a significantly elevated hazard of death with increasing age at diagnosis (HR 1.06, 1.03–1.08, *p*<0.001), liver disease (HR 2.17, 1.12–4.21, *p*=0.02), ILD (HR 2.14, 1.09–4.21, *p*=0.03), and inflammatory arthritis (HR 2.25, 1.05–4.80, *p*=0.04). An era effect was also evident (Table 5).

**TABLE 5:** Cox-proportional hazard models for the risk of death in the whole population of CVID patients showing only factors found to be significant on univariate analysis

	Univariate				Multivariate			
		95% CI				95% CI		
	HR	Lower	Upper	*p*	HR	Lower	Upper	*p*
Age at CVID diagnosis (y)	**1.06**	**1.03**	**1.08**	**<0.001**	**1.06**	**1.03**	**1.09**	**<0.001**
Era of diagnosis								
‘60–’79	**0.09**	**0.02**	**0.42**	**0.002**	**0.17**	**0.03**	**0.89**	**0.04**
** **‘80–’99	0.42	0.16	1.07	—	0.54	0.20	1.50	—
** **‘00–	1.00	—	—	—	1.00	—	—	—
Liver disease	**2.17**	**1.12**	**4.21**	**0.02**	**2.24**	**1.06**	**4.74**	**0.04**
Granulomatous lymphocytic interstitial lung disease	**2.14**	**1.09**	**4.21**	**0.03**	1.25	0.56	2.80	0.58
Inflammatory arthritis	**2.25**	**1.05**	**4.80**	**0.04**	1.95	0.88	4.34	0.10
Exposure to corticosteroids	1.80	0.94	3.46	0.08	1.80	0.84	3.84	0.13

Note: All bold values have a *p* value in the table.

Abbreviation: CVID, common variable immunodeficiency disorder.

Multivariate analysis confirmed the associations of age and earliest era of diagnosis, but not ILD or inflammatory arthritis, with the hazard of death. The presence of liver disease remained significantly associated with an elevated hazard of death in multivariate analysis (HR 2.24, 1.06–4.74, *p*=0.04) (Table 5). Exclusion of patients diagnosed in the 1960s and 1970s did not influence the results, with liver disease and age at diagnosis remaining the only factors associated with mortality (Supplemental Table S8, http://links.lww.com/HC9/A670).

**FIGURE 3:**
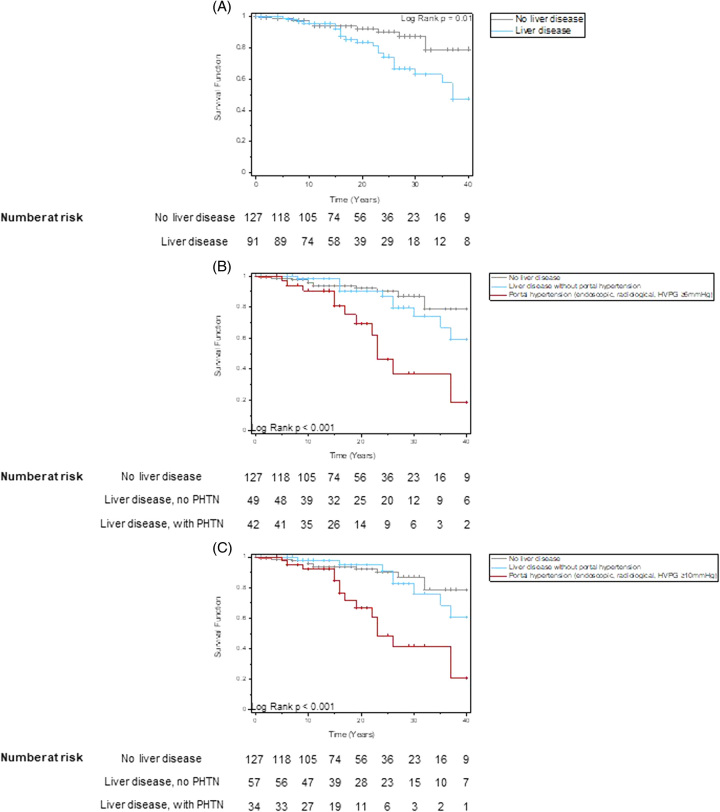
Kaplan-Meier curves for all-cause mortality comparing patients with and without CVID- related liver disease (A) and patients with PHTN, liver disease without PHTN, and those without liver disease (B and C), where PHTN was defined as the presence of radiological or endoscopic evidence of PHTN or HVPG (B) ≥6 mm Hg, (C) ≥10 mm Hg. Abbreviation: CVID, common variable immunodeficiency disorder; PHTN, portal hypertension.

Unadjusted Kaplan-Meier analysis demonstrated significantly impaired survival for patients with liver disease compared to those without (Figure 3A) with divergence from ~15 years following diagnosis of CVID. Likewise, survival was significantly lower for patients with PHTN (defined by the composite end points) compared to those with liver disease without PHTN and those without liver disease (Figure 3B, C).

### Factors associated with mortality in CVID-rLD

Univariate Cox-proportional hazard models for the risk of death in patients with CVID-rLD demonstrated that elevated liver stiffness was associated with an increased hazard of death (HR 1.05, 95%, CI: 1.01–1.08, *p*=0.006). Similarly, HVPG, the presence of radiological or endoscopic stigmata of PHTN, composite measure of PHTN, and histological evidence of stage 4 fibrosis were also significantly associated with an elevated hazard of death (Table 6). Multivariate analysis was not undertaken as few patients had all modalities of assessment performed.

**TABLE 6:** Univariate Cox-proportionate hazard models for mortality in patients with CVID-related liver disease.

			95% CI		
	Person years observed	HR for mortality	Lower	Upper	*p*
LSM (kPa) n=40	**104**	**1.05**	**1.01**	**1.08**	**0.006**
HVPG (mm Hg) n=36	**174**	**1.38**	**1.10**	**1.75**	**0.006**
Biopsy features: n=47 NRH	234	3.04	0.93	9.9	0.07
Granulomas	—	1.58	0.49	5.15	0.45
Fibrosis stage					
** **0–1	—	**1**	—	—	—
** **2	—	1.15	0.22	6.14	0.87
** **3	—	0.45	0.09	2.38	0.35
** **4	—	**9.53**	**1.14**	**79.56**	**0.04**
Biliary features	—	2.38	0.82	6.86	0.11
Steatosis	—	0.03	0	7.57	0.22
Inflammatory infiltrate	—	2.33	0.52	10.52	0.27
Evidence of PHTN					
Radiological n=90	**775**	**3.00**	**1.32**	**6.65**	**0.008**
Endoscopic n=62	**422**	**3.69**	**1.50**	**9.33**	**0.006**
HVPG ≥ 6 n=36	174	3.68	0.75	18.21	0.11
HVPG ≥ 10 n=36	174	2.51	0.413	15.22	0.32
Composite PHTN—endoscopic/radiological/HVPG n=91 (mm Hg)					
≥6	**862**	**3.72**	**1.60**	**8.64**	**0.002**
≥10	**862**	**3.45**	**1.52**	**7.81**	**0.003**

Note: All bold values have a *p* value in the table.

*Note:* n relates to number of patients assessed with the modality stated (fibrosis stage could only be assessed on 43 biopsies).

Abbreviation: CVID, common variable immunodeficiency disorder; LSM, liver stiffness measurement; NRH, nodular regenerative hyperplasia; PHTN, portal hypertension.

## DISCUSSION

In this large, single-center cohort of unselected patients with CVID, we have demonstrated that CVID-rLD is common, occurring in 42% of patients. Patients with CVID-rLD were more likely to have ILD, splenomegaly, enteropathy, and idiopathic thrombocytopenic purpura, demonstrating that CVID-rLD is part of a multisystem disorder in complex CVID.

The presence of liver disease was not significantly associated with age at diagnosis of CVID, confirming other reports,^8^ nor with sex. CVID-rLD occurs early in the natural history of CVID, with median onset at 6 years from diagnosis and onset of PHTN at 10.5 years from diagnosis, similar to other cohorts.^9^ PHTN was a common complication of CVID-rLD, occurring in 44% of patients with CVID-rLD. This frequency is similar to patients with NRH (31%–55%) and with other reports of CVID-rLD (21%–75%)^2,6,7,12,17,18^ and demonstrates that CVID-rLD is associated with significant clinical sequalae.

The epidemiology and natural history of liver disease in CVID have been challenging to define due to the changing etiology as the risk of viral hepatitis receded and changing patterns of screening as the importance of liver disease has been recognized. The published prevalence of liver disease in patients with CVID ranges from 5% to 79%,^2–4,7–12^ and our estimate of 42% sits within the middle of this range. By including all patients with CVID from our center, who were well screened (LFTs measured in all and abdominal imaging in 94%) this is likely a reasonable estimate of the prevalence in this tertiary patient cohort.

NRH-LC was a common histological feature in CVID-rLD, seen in nearly two-thirds of liver biopsies performed in this group of patients and granulomas in nearly a quarter. Advanced fibrosis was frequently observed, with stage 3 or above seen in over half of all biopsies. This fits with other cohorts describing cirrhosis (equivalent to stage 4 in the scoring system used here) in one-third of patients with NRH-LC.^7^ The association of elevated LSM with NRH-LC and PHTN^9,15^ may reflect the attendant fibrosis seen with NRH-LC in CVID.

Liver disease was associated with an elevated crude mortality rate (26.4% vs. 11%) and an adjusted HR of 2.24 (*p*=0.04) for mortality, similar to other reports (HR 2.48–3.5).^7,13^ The presence of PHTN, elevated LSM, and advanced fibrosis were all associated with poorer survival and an elevated hazard of death. In addition, we have demonstrated that after correction for liver disease, other organ involvement was not independently associated with mortality, suggesting that liver disease may be a significant factor in patient mortality with CVID. While understanding the causes of death would have been helpful, we were unable to reliably obtain this as deaths often occurred at other locations due to the large referral area for our service. However, the high frequency of decompensated liver disease suggests that liver disease complications are not uncommon for these patients. Variceal hemorrhage, ascites, spontaneous bacterial peritonitis, HE, hepatopulmonary syndrome, and liver transplantation are all recognized outcomes for patients with CVID.^2,19,20^ Therefore, as CVID-rLD is associated with end-stage liver disease and mortality, it is likely CVID-rLD not only represents a severe phenotype of CVID, but also contributes to mortality.

LSM is a valuable tool in the assessment of CVID-rLD. Elevated LSM is associated with liver enzyme abnormalities, NRH-LC, PHTN, and enteropathy in CVID, with cutoffs of 6.2 and 11.2 kPa reported to detect NRH-LC and PHTN, respectively.^9,10,15^ We observed higher LSM in those with evidence of PHTN, although a low value did not exclude PHTN. Therefore, elevation in LSM should prompt assessment for further evidence of liver disease and PHTN; however, a normal LSM does not exclude significant pathology. In non-CVID–related NRH, a correlation between LSM and PHTN has not been observed,^18^ which suggests that CVID-rLD may have different characteristics compared to NRH in other settings. Due to the different mechanisms of PHTN compared to cirrhosis, conventional noninvasive thresholds for the stratification of endoscopic surveillance are inappropriate for CVID^14^ even though patients may develop cirrhosis. In CVID, endoscopic screening should be considered in all patients with LSM of >6.2 kPa or when clinical or radiological stigmata of PHTN are evident.

Overall, the performance of HVPG to predict clinical evidence of PHTN was poor, likely because NRH is associated with presinusoidal PHTN, which is not reflected in the HVPG. However, our cohort with HVPG measurements was small, so these observations must be treated with caution. In NRH, the HVPG is typically modestly elevated (7–9 mm Hg), which is significantly lower than directly measured portal pressure gradients.^21,22^ It is possible that those with an elevated HVPG and NRH-LC may have both sinusoidal and presinusoidal contributions to PHTN, raising the question of whether this subgroup is at higher risk of complications.

The pathogenesis of CVID-rLD remains undefined. NRH is a secondary phenomenon that occurs in a range of conditions, including drug exposure, chronic infection, prothrombotic states, and immunodeficiency. It may result from intrahepatic vascular insults resulting in regional atrophy and adjacent nodular regeneration with additional vascular compression.^23–25^ Liver-specific, clonally expanded intrasinusoidal CD8 T cells localized to areas of LSEC damage,^26^ endothelitis^27^ and altered immunoregulatory gene expression and cytokine synthesis^11,28^ have been reported suggesting a role for immune-mediated vascular injury. We recently reported changes of capillarisation in the liver sinusoidal endothelium, providing direct evidence of vascular changes in CVID-rLD.^29^ Lymphocytes have also been observed at sites of focal hepatocyte damage in CVID^12^ raising the possibility of additional immune-mediated parenchymal injury. Whether these phenomena occur due to intrinsic immune dysregulation in CVID or secondary to other phenomena remains unknown. For example, increased splenic vein blood flow secondary to splenomegaly may alter hemodynamics,^30^ conceivably influencing LSEC. Additionally, reductions in mucosal antibody and GI inflammation, infection and dysbiosis may result in microbial translocation, leading to damage or inflammatory responses within the liver.^31,32^

We observed a significant association between GI involvement and CVID-rLD, as have others,^8–10,12^ although whether this reflects a causal relationship or simply evidence for a multisystem disorder remains to be established. We could not identify an association between small bowel villous blunting and CVID-rLD, but only a minority had duodenal biopsies and these were targeted to those with symptoms, so were not representative of the whole CVID cohort. However, other groups have reported such an association.^12^

This study reports a high rate of liver disease and PHTN in a large cohort with long duration of follow-up. However, this may be an underestimate as comprehensive evaluation was only undertaken when there was clinical suspicion of liver disease rather than in an unselected fashion. Due to the retrospective nature, missing data and failure to identify patients in older cohorts may have introduced bias. Additionally, we cannot ascribe mortality to liver disease as cause of death data was not available. The lower mortality in the earliest era may be explained by factors including survivorship bias, as those who died early may have been missed from the cohort. Additionally, increasing referral of patients with complex phenotypes in recent years may have resulted in our cohort becoming higher risk with time. Finally, the exclusion of those with viral hepatitis would have disproportionally affected earlier patients. However, no significant differences were seen following the exclusion of the earliest cohort, suggesting our findings are robust. While not all patients with liver disease had all investigations performed, there were no unexpected differences between those who had LSM, endoscopy, liver biopsy, or HVPG measurement compared to those who did not, suggesting the findings are generalizable.

The cornerstone of current management for CVID is the use of polyvalent IgG replacement that reduces infectious complications and improves prognosis.^1,33^ However, no treatments have been shown to improve outcomes for immune-mediated complications of CVID, including CVID-rLD. This, coupled with poor published post-liver transplant outcomes^19^, means there is a significant unmet need for treatments.

We have demonstrated in a large cohort of patients with CVID with long follow-up that CVID-rLD was a common complication that begins early in the disease course. Patients with liver disease were more likely to have other complications, suggesting that CVID-rLD is part of a complex, multisystem disorder. PHTN was common in patients with liver disease, occurring in 44%, and liver disease and PHTN were associated with an increased hazard of mortality. Liver disease was the only end-organ disease associated with mortality in adjusted analyses.

Evidence of CVID-rLD should be actively sought in all patients with CVID. We recommend screening all patients with CVID for liver disease with at least annual LFTs and regular abdominal imaging and LSM. Clinicians should have a low threshold for cross-sectional abdominal imaging, endoscopy, and liver biopsy to allow for prognostication and pre-emptive management of PHTN, especially in those with features of complex CVID. There remain significant unmet needs including treatments for CVID-rLD, strategies and biomarkers to identify high-risk patients and understanding of the pathogenesis and causes of death in patients with CVID-rLD.

## Supplementary Material

SUPPLEMENTARY MATERIAL
