# Supplementary material for: Common variable immunodeficiency disorder-related liver disease is common and results in portal hypertension and an increased risk of death
Source: Hepatol Commun. 2023 Dec 15;8(1):e0322. doi: 10.1097/HC9.0000000000000322 (PMC10727572; doi:10.1097/HC9.0000000000000322)
Supplement: SUPPLEMENTARY MATERIAL [file hc9-8-e0322-s001.pptx]

## Slide 1
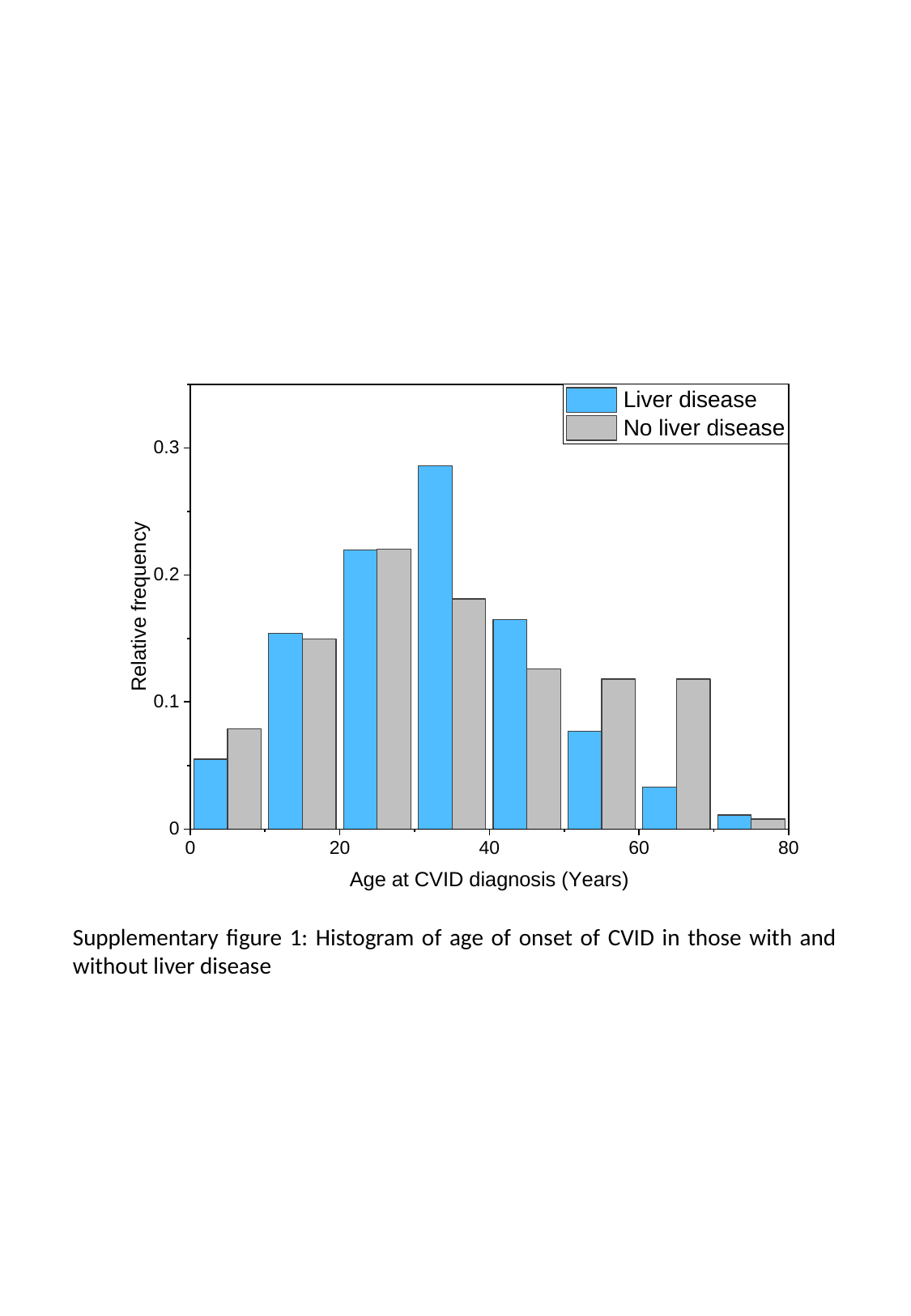

Supplementary figure 1: Histogram of age of onset of CVID in those with and without liver disease

## Slide 2
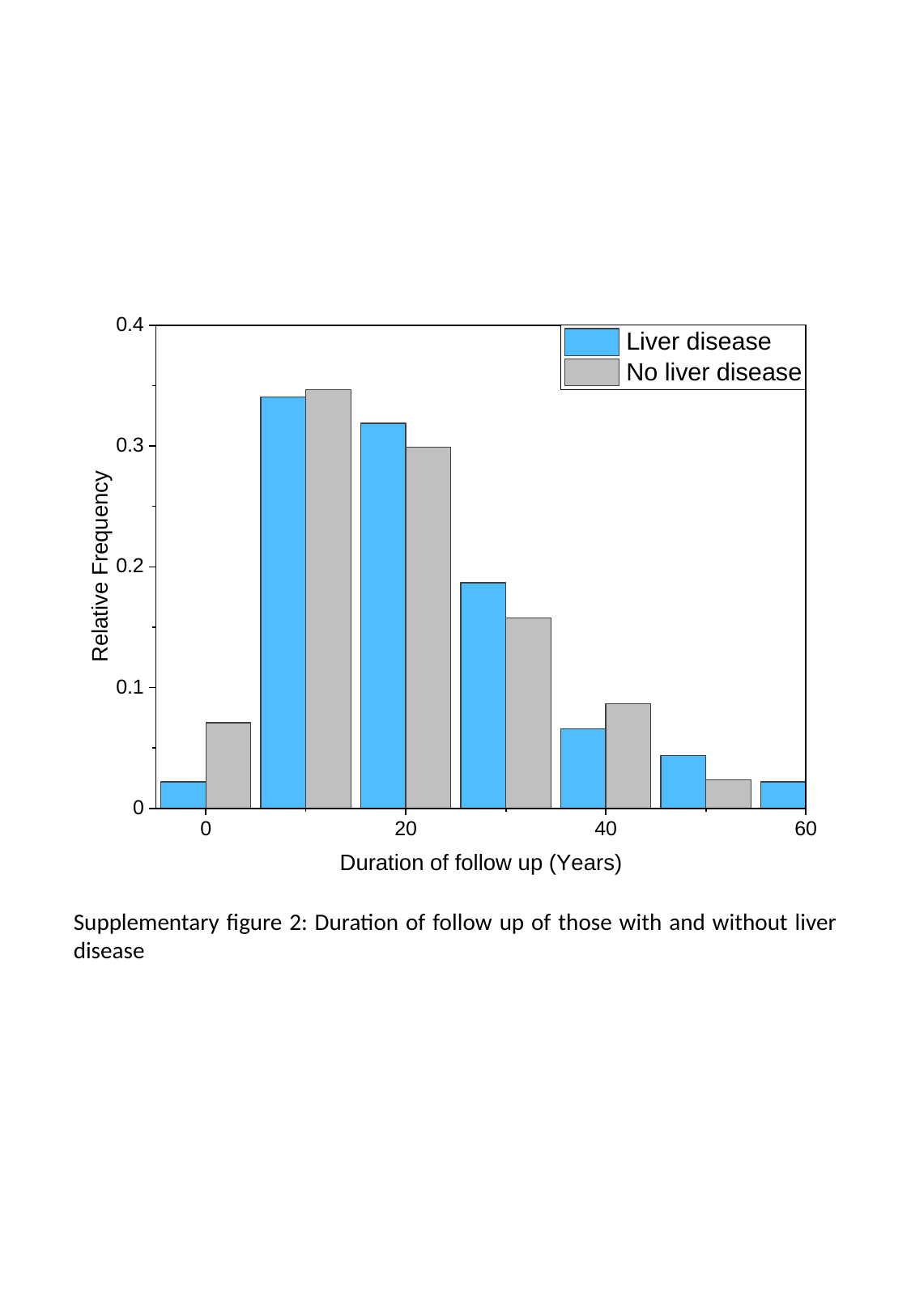

Supplementary figure 2: Duration of follow up of those with and without liver disease

## Slide 3
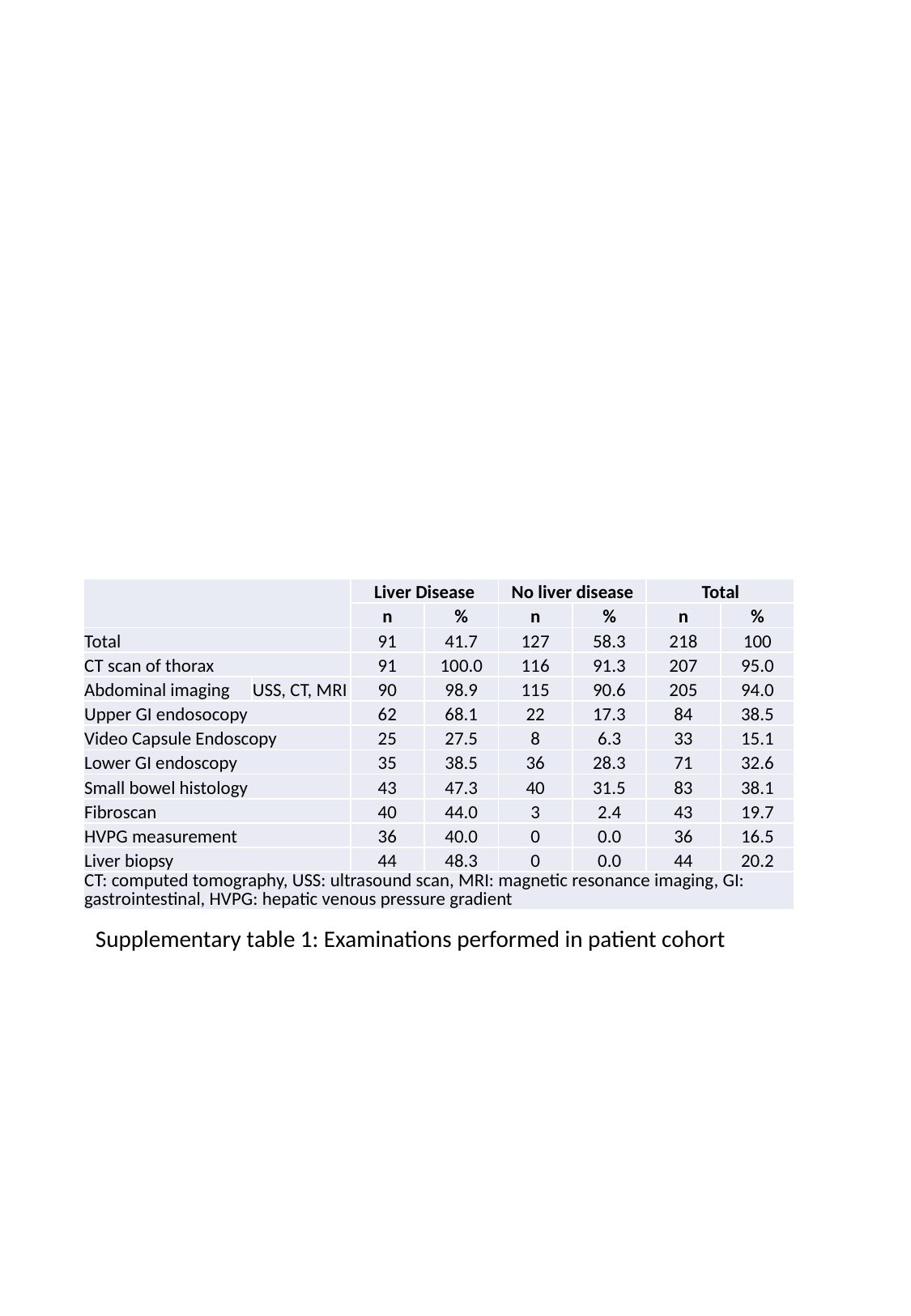

| | | Liver Disease | | No liver disease | | Total | |
| --- | --- | --- | --- | --- | --- | --- | --- |
| | | n | % | n | % | n | % |
| Total | | 91 | 41.7 | 127 | 58.3 | 218 | 100 |
| CT scan of thorax | | 91 | 100.0 | 116 | 91.3 | 207 | 95.0 |
| Abdominal imaging | USS, CT, MRI | 90 | 98.9 | 115 | 90.6 | 205 | 94.0 |
| Upper GI endosocopy | | 62 | 68.1 | 22 | 17.3 | 84 | 38.5 |
| Video Capsule Endoscopy | | 25 | 27.5 | 8 | 6.3 | 33 | 15.1 |
| Lower GI endoscopy | | 35 | 38.5 | 36 | 28.3 | 71 | 32.6 |
| Small bowel histology | | 43 | 47.3 | 40 | 31.5 | 83 | 38.1 |
| Fibroscan | | 40 | 44.0 | 3 | 2.4 | 43 | 19.7 |
| HVPG measurement | | 36 | 40.0 | 0 | 0.0 | 36 | 16.5 |
| Liver biopsy | | 44 | 48.3 | 0 | 0.0 | 44 | 20.2 |
| CT: computed tomography, USS: ultrasound scan, MRI: magnetic resonance imaging, GI: gastrointestinal, HVPG: hepatic venous pressure gradient | | | | | | | |
Supplementary table 1: Examinations performed in patient cohort

## Slide 4
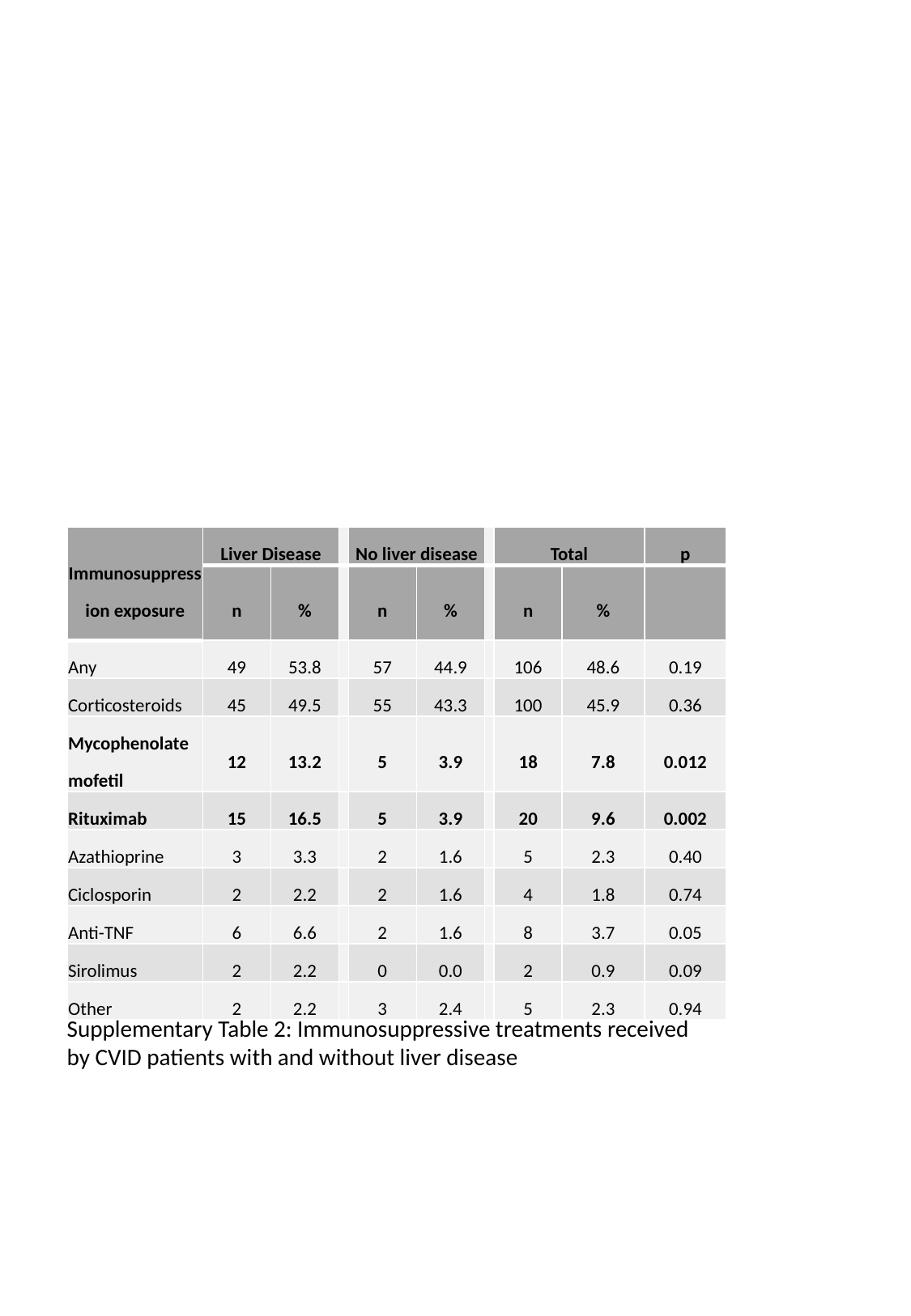

| Immunosuppression exposure | Liver Disease | | | No liver disease | | | Total | | p |
| --- | --- | --- | --- | --- | --- | --- | --- | --- | --- |
| | n | % | | n | % | | n | % | |
| Any | 49 | 53.8 | | 57 | 44.9 | | 106 | 48.6 | 0.19 |
| Corticosteroids | 45 | 49.5 | | 55 | 43.3 | | 100 | 45.9 | 0.36 |
| Mycophenolate mofetil | 12 | 13.2 | | 5 | 3.9 | | 18 | 7.8 | 0.012 |
| Rituximab | 15 | 16.5 | | 5 | 3.9 | | 20 | 9.6 | 0.002 |
| Azathioprine | 3 | 3.3 | | 2 | 1.6 | | 5 | 2.3 | 0.40 |
| Ciclosporin | 2 | 2.2 | | 2 | 1.6 | | 4 | 1.8 | 0.74 |
| Anti-TNF | 6 | 6.6 | | 2 | 1.6 | | 8 | 3.7 | 0.05 |
| Sirolimus | 2 | 2.2 | | 0 | 0.0 | | 2 | 0.9 | 0.09 |
| Other | 2 | 2.2 | | 3 | 2.4 | | 5 | 2.3 | 0.94 |
Supplementary Table 2: Immunosuppressive treatments received by CVID patients with and without liver disease

## Slide 5
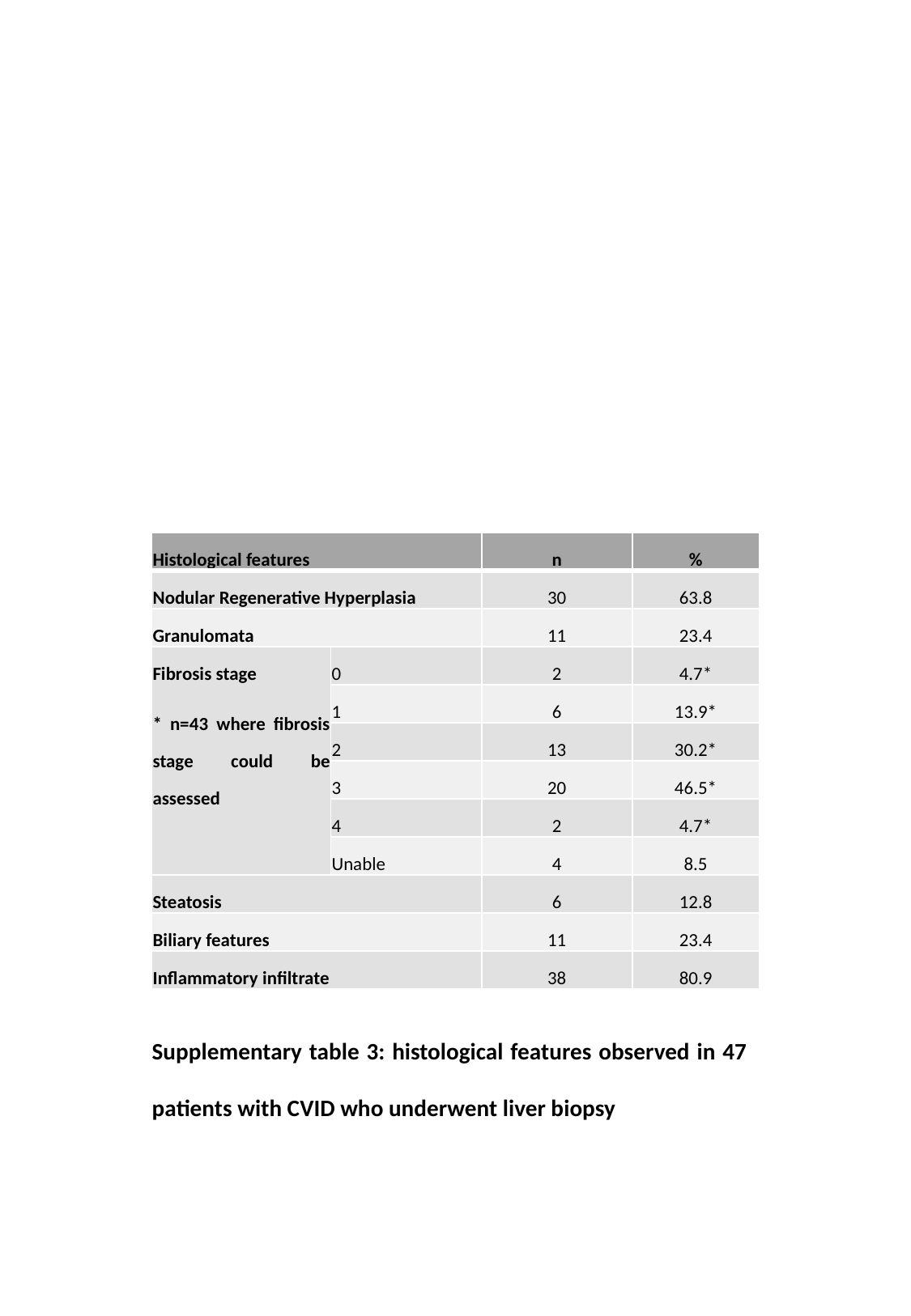

#
| Histological features | | n | % |
| --- | --- | --- | --- |
| Nodular Regenerative Hyperplasia | | 30 | 63.8 |
| Granulomata | | 11 | 23.4 |
| Fibrosis stage \* n=43 where fibrosis stage could be assessed | 0 | 2 | 4.7\* |
| | 1 | 6 | 13.9\* |
| | 2 | 13 | 30.2\* |
| | 3 | 20 | 46.5\* |
| | 4 | 2 | 4.7\* |
| | Unable | 4 | 8.5 |
| Steatosis | | 6 | 12.8 |
| Biliary features | | 11 | 23.4 |
| Inflammatory infiltrate | | 38 | 80.9 |
Supplementary table 3: histological features observed in 47 patients with CVID who underwent liver biopsy

## Slide 6
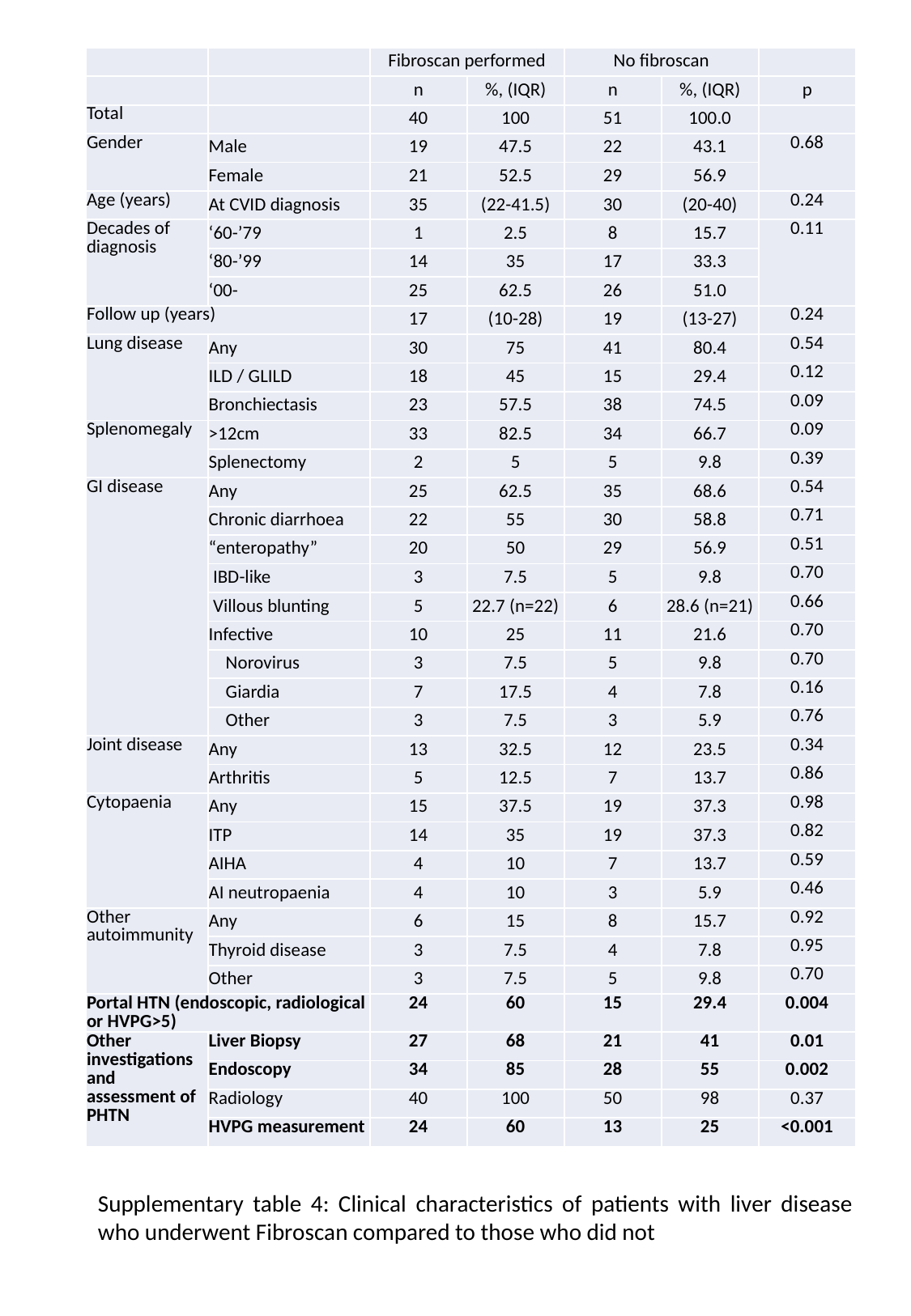

| | | Fibroscan performed | | No fibroscan | | |
| --- | --- | --- | --- | --- | --- | --- |
| | | n | %, (IQR) | n | %, (IQR) | p |
| Total | | 40 | 100 | 51 | 100.0 | |
| Gender | Male | 19 | 47.5 | 22 | 43.1 | 0.68 |
| | Female | 21 | 52.5 | 29 | 56.9 | |
| Age (years) | At CVID diagnosis | 35 | (22-41.5) | 30 | (20-40) | 0.24 |
| Decades of diagnosis | ‘60-’79 | 1 | 2.5 | 8 | 15.7 | 0.11 |
| | ‘80-’99 | 14 | 35 | 17 | 33.3 | |
| | ‘00- | 25 | 62.5 | 26 | 51.0 | |
| Follow up (years) | | 17 | (10-28) | 19 | (13-27) | 0.24 |
| Lung disease | Any | 30 | 75 | 41 | 80.4 | 0.54 |
| | ILD / GLILD | 18 | 45 | 15 | 29.4 | 0.12 |
| | Bronchiectasis | 23 | 57.5 | 38 | 74.5 | 0.09 |
| Splenomegaly | >12cm | 33 | 82.5 | 34 | 66.7 | 0.09 |
| | Splenectomy | 2 | 5 | 5 | 9.8 | 0.39 |
| GI disease | Any | 25 | 62.5 | 35 | 68.6 | 0.54 |
| | Chronic diarrhoea | 22 | 55 | 30 | 58.8 | 0.71 |
| | “enteropathy” | 20 | 50 | 29 | 56.9 | 0.51 |
| | IBD-like | 3 | 7.5 | 5 | 9.8 | 0.70 |
| | Villous blunting | 5 | 22.7 (n=22) | 6 | 28.6 (n=21) | 0.66 |
| | Infective | 10 | 25 | 11 | 21.6 | 0.70 |
| | Norovirus | 3 | 7.5 | 5 | 9.8 | 0.70 |
| | Giardia | 7 | 17.5 | 4 | 7.8 | 0.16 |
| | Other | 3 | 7.5 | 3 | 5.9 | 0.76 |
| Joint disease | Any | 13 | 32.5 | 12 | 23.5 | 0.34 |
| | Arthritis | 5 | 12.5 | 7 | 13.7 | 0.86 |
| Cytopaenia | Any | 15 | 37.5 | 19 | 37.3 | 0.98 |
| | ITP | 14 | 35 | 19 | 37.3 | 0.82 |
| | AIHA | 4 | 10 | 7 | 13.7 | 0.59 |
| | AI neutropaenia | 4 | 10 | 3 | 5.9 | 0.46 |
| Other autoimmunity | Any | 6 | 15 | 8 | 15.7 | 0.92 |
| | Thyroid disease | 3 | 7.5 | 4 | 7.8 | 0.95 |
| | Other | 3 | 7.5 | 5 | 9.8 | 0.70 |
| Portal HTN (endoscopic, radiological or HVPG>5) | | 24 | 60 | 15 | 29.4 | 0.004 |
| Other investigations and assessment of PHTN | Liver Biopsy | 27 | 68 | 21 | 41 | 0.01 |
| | Endoscopy | 34 | 85 | 28 | 55 | 0.002 |
| | Radiology | 40 | 100 | 50 | 98 | 0.37 |
| | HVPG measurement | 24 | 60 | 13 | 25 | <0.001 |
Supplementary table 4: Clinical characteristics of patients with liver disease who underwent Fibroscan compared to those who did not

## Slide 7
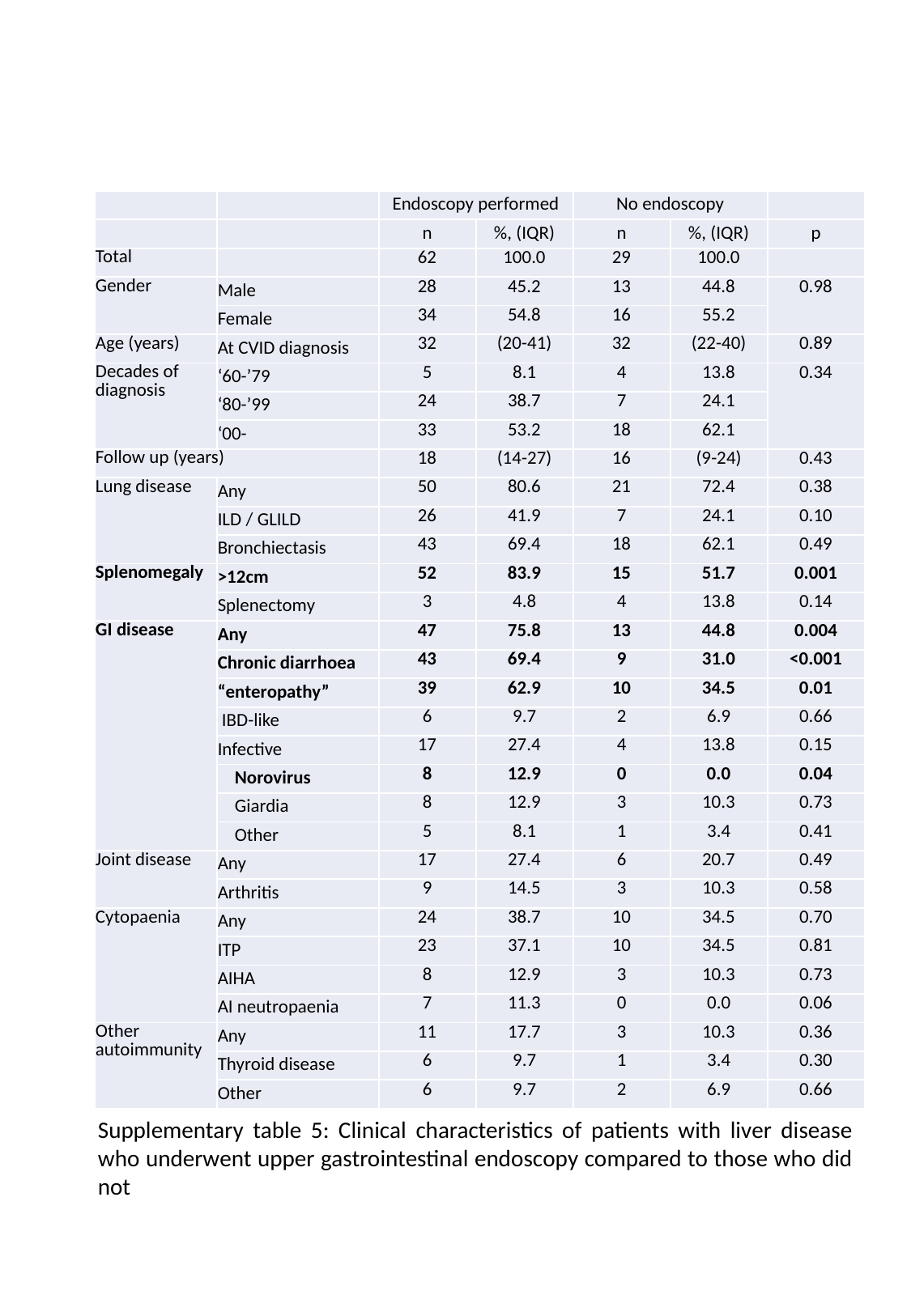

| | | Endoscopy performed | | No endoscopy | | |
| --- | --- | --- | --- | --- | --- | --- |
| | | n | %, (IQR) | n | %, (IQR) | p |
| Total | | 62 | 100.0 | 29 | 100.0 | |
| Gender | Male | 28 | 45.2 | 13 | 44.8 | 0.98 |
| | Female | 34 | 54.8 | 16 | 55.2 | |
| Age (years) | At CVID diagnosis | 32 | (20-41) | 32 | (22-40) | 0.89 |
| Decades of diagnosis | ‘60-’79 | 5 | 8.1 | 4 | 13.8 | 0.34 |
| | ‘80-’99 | 24 | 38.7 | 7 | 24.1 | |
| | ‘00- | 33 | 53.2 | 18 | 62.1 | |
| Follow up (years) | | 18 | (14-27) | 16 | (9-24) | 0.43 |
| Lung disease | Any | 50 | 80.6 | 21 | 72.4 | 0.38 |
| | ILD / GLILD | 26 | 41.9 | 7 | 24.1 | 0.10 |
| | Bronchiectasis | 43 | 69.4 | 18 | 62.1 | 0.49 |
| Splenomegaly | >12cm | 52 | 83.9 | 15 | 51.7 | 0.001 |
| | Splenectomy | 3 | 4.8 | 4 | 13.8 | 0.14 |
| GI disease | Any | 47 | 75.8 | 13 | 44.8 | 0.004 |
| | Chronic diarrhoea | 43 | 69.4 | 9 | 31.0 | <0.001 |
| | “enteropathy” | 39 | 62.9 | 10 | 34.5 | 0.01 |
| | IBD-like | 6 | 9.7 | 2 | 6.9 | 0.66 |
| | Infective | 17 | 27.4 | 4 | 13.8 | 0.15 |
| | Norovirus | 8 | 12.9 | 0 | 0.0 | 0.04 |
| | Giardia | 8 | 12.9 | 3 | 10.3 | 0.73 |
| | Other | 5 | 8.1 | 1 | 3.4 | 0.41 |
| Joint disease | Any | 17 | 27.4 | 6 | 20.7 | 0.49 |
| | Arthritis | 9 | 14.5 | 3 | 10.3 | 0.58 |
| Cytopaenia | Any | 24 | 38.7 | 10 | 34.5 | 0.70 |
| | ITP | 23 | 37.1 | 10 | 34.5 | 0.81 |
| | AIHA | 8 | 12.9 | 3 | 10.3 | 0.73 |
| | AI neutropaenia | 7 | 11.3 | 0 | 0.0 | 0.06 |
| Other autoimmunity | Any | 11 | 17.7 | 3 | 10.3 | 0.36 |
| | Thyroid disease | 6 | 9.7 | 1 | 3.4 | 0.30 |
| | Other | 6 | 9.7 | 2 | 6.9 | 0.66 |
Supplementary table 5: Clinical characteristics of patients with liver disease who underwent upper gastrointestinal endoscopy compared to those who did not

## Slide 8
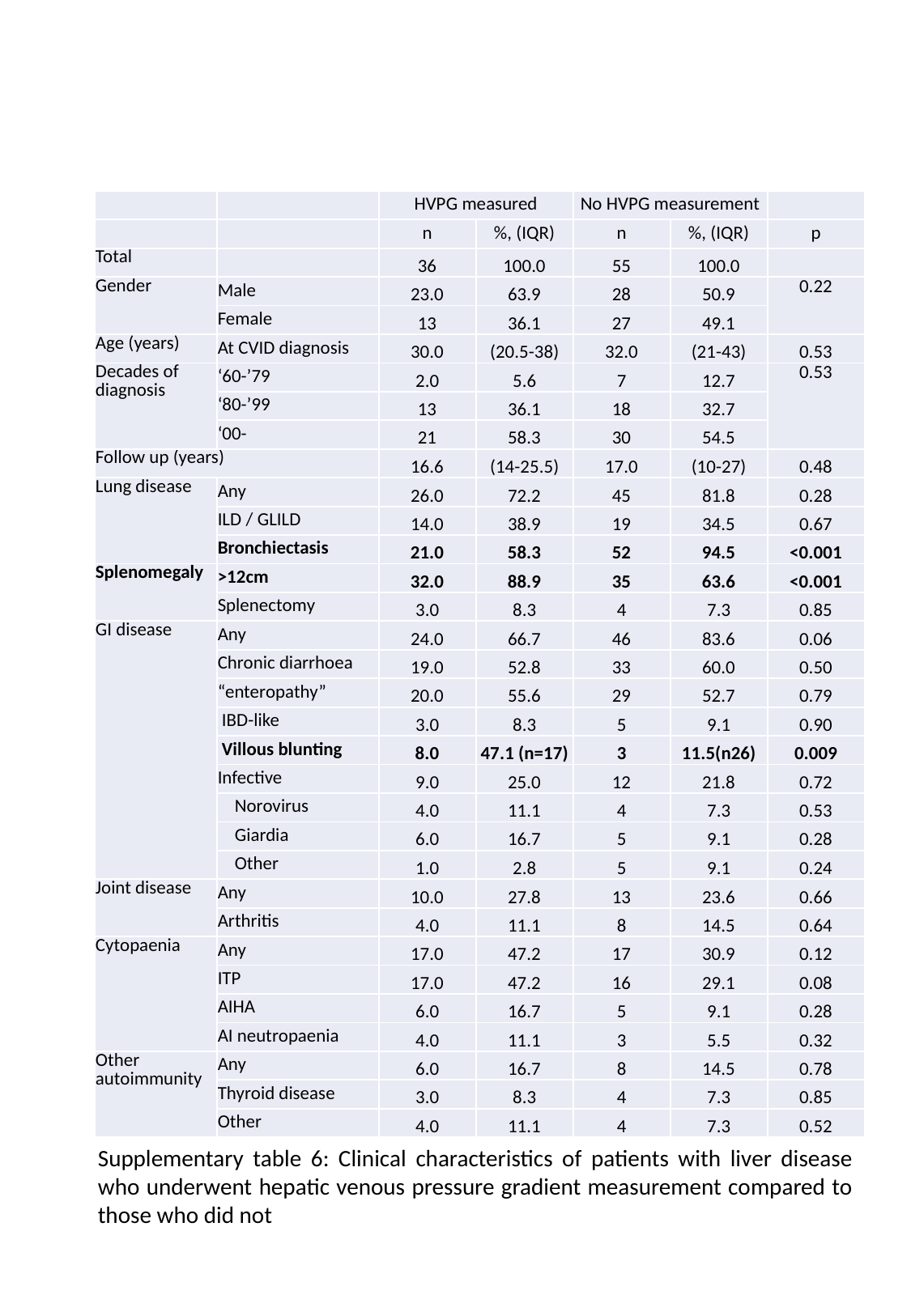

| | | HVPG measured | | No HVPG measurement | | |
| --- | --- | --- | --- | --- | --- | --- |
| | | n | %, (IQR) | n | %, (IQR) | p |
| Total | | 36 | 100.0 | 55 | 100.0 | |
| Gender | Male | 23.0 | 63.9 | 28 | 50.9 | 0.22 |
| | Female | 13 | 36.1 | 27 | 49.1 | |
| Age (years) | At CVID diagnosis | 30.0 | (20.5-38) | 32.0 | (21-43) | 0.53 |
| Decades of diagnosis | ‘60-’79 | 2.0 | 5.6 | 7 | 12.7 | 0.53 |
| | ‘80-’99 | 13 | 36.1 | 18 | 32.7 | |
| | ‘00- | 21 | 58.3 | 30 | 54.5 | |
| Follow up (years) | | 16.6 | (14-25.5) | 17.0 | (10-27) | 0.48 |
| Lung disease | Any | 26.0 | 72.2 | 45 | 81.8 | 0.28 |
| | ILD / GLILD | 14.0 | 38.9 | 19 | 34.5 | 0.67 |
| | Bronchiectasis | 21.0 | 58.3 | 52 | 94.5 | <0.001 |
| Splenomegaly | >12cm | 32.0 | 88.9 | 35 | 63.6 | <0.001 |
| | Splenectomy | 3.0 | 8.3 | 4 | 7.3 | 0.85 |
| GI disease | Any | 24.0 | 66.7 | 46 | 83.6 | 0.06 |
| | Chronic diarrhoea | 19.0 | 52.8 | 33 | 60.0 | 0.50 |
| | “enteropathy” | 20.0 | 55.6 | 29 | 52.7 | 0.79 |
| | IBD-like | 3.0 | 8.3 | 5 | 9.1 | 0.90 |
| | Villous blunting | 8.0 | 47.1 (n=17) | 3 | 11.5(n26) | 0.009 |
| | Infective | 9.0 | 25.0 | 12 | 21.8 | 0.72 |
| | Norovirus | 4.0 | 11.1 | 4 | 7.3 | 0.53 |
| | Giardia | 6.0 | 16.7 | 5 | 9.1 | 0.28 |
| | Other | 1.0 | 2.8 | 5 | 9.1 | 0.24 |
| Joint disease | Any | 10.0 | 27.8 | 13 | 23.6 | 0.66 |
| | Arthritis | 4.0 | 11.1 | 8 | 14.5 | 0.64 |
| Cytopaenia | Any | 17.0 | 47.2 | 17 | 30.9 | 0.12 |
| | ITP | 17.0 | 47.2 | 16 | 29.1 | 0.08 |
| | AIHA | 6.0 | 16.7 | 5 | 9.1 | 0.28 |
| | AI neutropaenia | 4.0 | 11.1 | 3 | 5.5 | 0.32 |
| Other autoimmunity | Any | 6.0 | 16.7 | 8 | 14.5 | 0.78 |
| | Thyroid disease | 3.0 | 8.3 | 4 | 7.3 | 0.85 |
| | Other | 4.0 | 11.1 | 4 | 7.3 | 0.52 |
Supplementary table 6: Clinical characteristics of patients with liver disease who underwent hepatic venous pressure gradient measurement compared to those who did not

## Slide 9
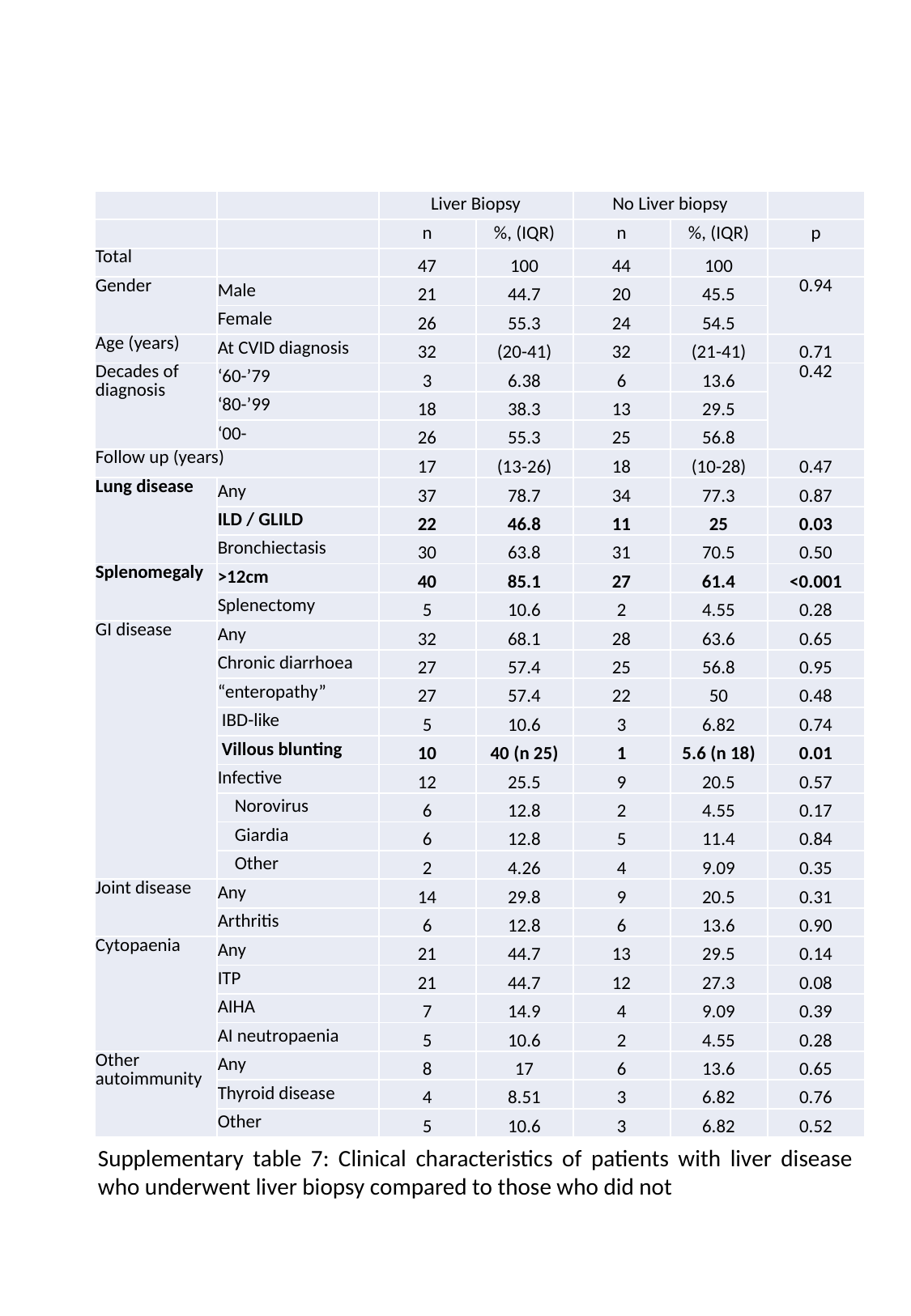

| | | Liver Biopsy | | No Liver biopsy | | |
| --- | --- | --- | --- | --- | --- | --- |
| | | n | %, (IQR) | n | %, (IQR) | p |
| Total | | 47 | 100 | 44 | 100 | |
| Gender | Male | 21 | 44.7 | 20 | 45.5 | 0.94 |
| | Female | 26 | 55.3 | 24 | 54.5 | |
| Age (years) | At CVID diagnosis | 32 | (20-41) | 32 | (21-41) | 0.71 |
| Decades of diagnosis | ‘60-’79 | 3 | 6.38 | 6 | 13.6 | 0.42 |
| | ‘80-’99 | 18 | 38.3 | 13 | 29.5 | |
| | ‘00- | 26 | 55.3 | 25 | 56.8 | |
| Follow up (years) | | 17 | (13-26) | 18 | (10-28) | 0.47 |
| Lung disease | Any | 37 | 78.7 | 34 | 77.3 | 0.87 |
| | ILD / GLILD | 22 | 46.8 | 11 | 25 | 0.03 |
| | Bronchiectasis | 30 | 63.8 | 31 | 70.5 | 0.50 |
| Splenomegaly | >12cm | 40 | 85.1 | 27 | 61.4 | <0.001 |
| | Splenectomy | 5 | 10.6 | 2 | 4.55 | 0.28 |
| GI disease | Any | 32 | 68.1 | 28 | 63.6 | 0.65 |
| | Chronic diarrhoea | 27 | 57.4 | 25 | 56.8 | 0.95 |
| | “enteropathy” | 27 | 57.4 | 22 | 50 | 0.48 |
| | IBD-like | 5 | 10.6 | 3 | 6.82 | 0.74 |
| | Villous blunting | 10 | 40 (n 25) | 1 | 5.6 (n 18) | 0.01 |
| | Infective | 12 | 25.5 | 9 | 20.5 | 0.57 |
| | Norovirus | 6 | 12.8 | 2 | 4.55 | 0.17 |
| | Giardia | 6 | 12.8 | 5 | 11.4 | 0.84 |
| | Other | 2 | 4.26 | 4 | 9.09 | 0.35 |
| Joint disease | Any | 14 | 29.8 | 9 | 20.5 | 0.31 |
| | Arthritis | 6 | 12.8 | 6 | 13.6 | 0.90 |
| Cytopaenia | Any | 21 | 44.7 | 13 | 29.5 | 0.14 |
| | ITP | 21 | 44.7 | 12 | 27.3 | 0.08 |
| | AIHA | 7 | 14.9 | 4 | 9.09 | 0.39 |
| | AI neutropaenia | 5 | 10.6 | 2 | 4.55 | 0.28 |
| Other autoimmunity | Any | 8 | 17 | 6 | 13.6 | 0.65 |
| | Thyroid disease | 4 | 8.51 | 3 | 6.82 | 0.76 |
| | Other | 5 | 10.6 | 3 | 6.82 | 0.52 |
Supplementary table 7: Clinical characteristics of patients with liver disease who underwent liver biopsy compared to those who did not

## Slide 10
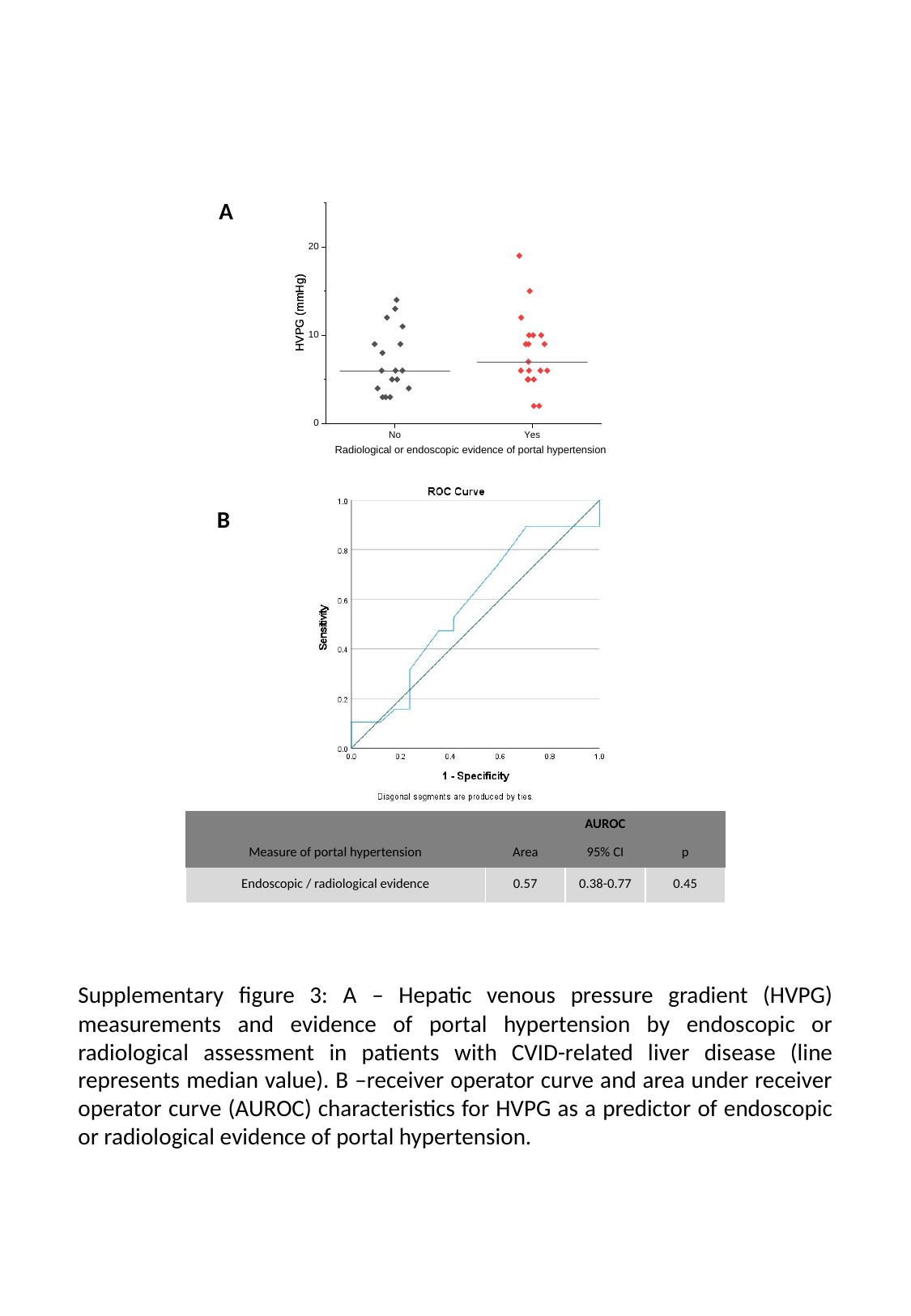

A
B
| | AUROC | AUROC | |
| --- | --- | --- | --- |
| Measure of portal hypertension | Area | 95% CI | p |
| Endoscopic / radiological evidence | 0.57 | 0.38-0.77 | 0.45 |
Supplementary figure 3: A – Hepatic venous pressure gradient (HVPG) measurements and evidence of portal hypertension by endoscopic or radiological assessment in patients with CVID-related liver disease (line represents median value). B –receiver operator curve and area under receiver operator curve (AUROC) characteristics for HVPG as a predictor of endoscopic or radiological evidence of portal hypertension.

## Slide 11
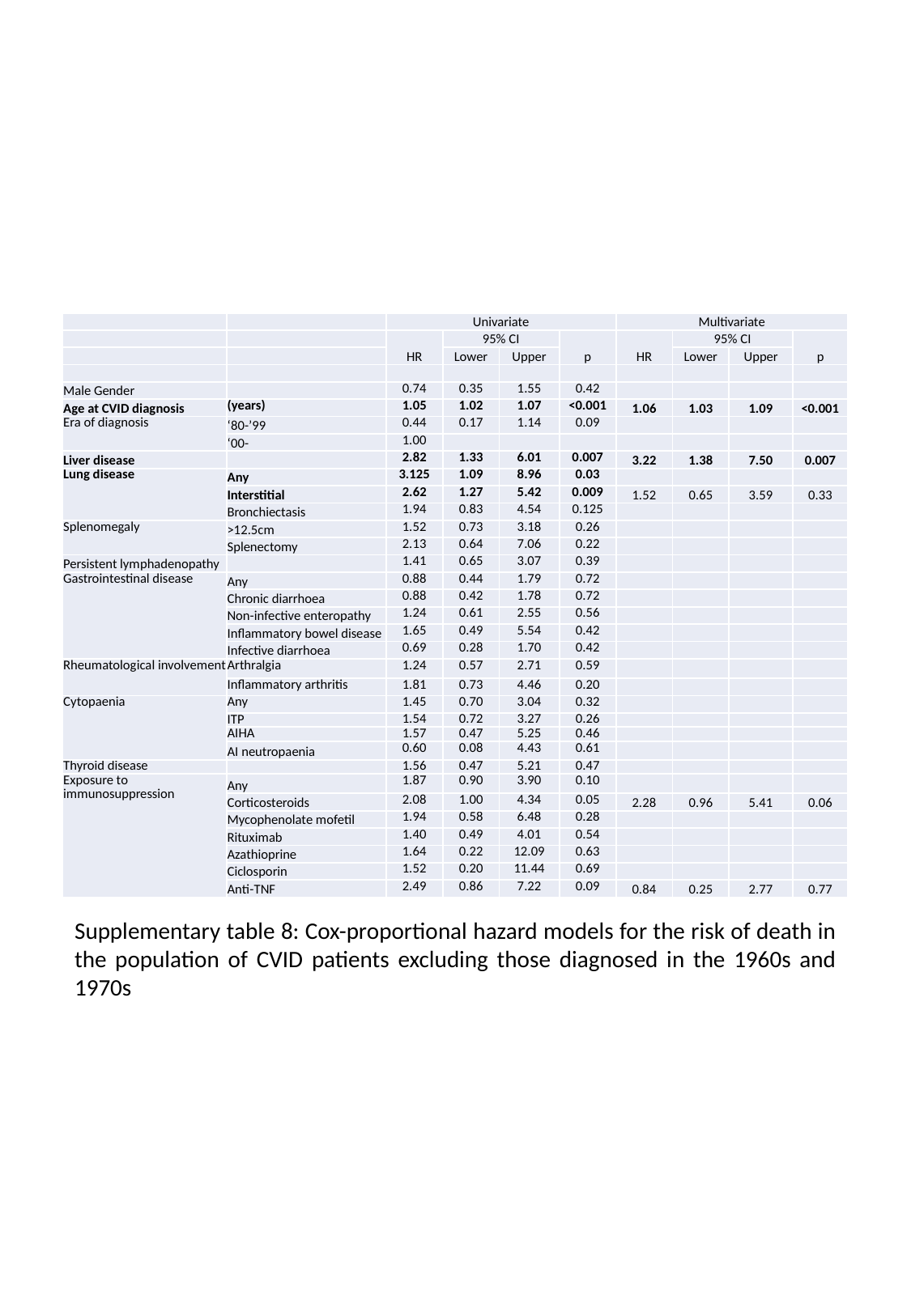

| | | Univariate | | | | Multivariate | | | |
| --- | --- | --- | --- | --- | --- | --- | --- | --- | --- |
| | | HR | 95% CI | | p | HR | 95% CI | | p |
| | | | Lower | Upper | | | Lower | Upper | |
| | | | | | | | | | |
| Male Gender | | 0.74 | 0.35 | 1.55 | 0.42 | | | | |
| Age at CVID diagnosis | (years) | 1.05 | 1.02 | 1.07 | <0.001 | 1.06 | 1.03 | 1.09 | <0.001 |
| Era of diagnosis | ‘80-’99 | 0.44 | 0.17 | 1.14 | 0.09 | | | | |
| | ‘00- | 1.00 | | | | | | | |
| Liver disease | | 2.82 | 1.33 | 6.01 | 0.007 | 3.22 | 1.38 | 7.50 | 0.007 |
| Lung disease | Any | 3.125 | 1.09 | 8.96 | 0.03 | | | | |
| | Interstitial | 2.62 | 1.27 | 5.42 | 0.009 | 1.52 | 0.65 | 3.59 | 0.33 |
| | Bronchiectasis | 1.94 | 0.83 | 4.54 | 0.125 | | | | |
| Splenomegaly | >12.5cm | 1.52 | 0.73 | 3.18 | 0.26 | | | | |
| | Splenectomy | 2.13 | 0.64 | 7.06 | 0.22 | | | | |
| Persistent lymphadenopathy | | 1.41 | 0.65 | 3.07 | 0.39 | | | | |
| Gastrointestinal disease | Any | 0.88 | 0.44 | 1.79 | 0.72 | | | | |
| | Chronic diarrhoea | 0.88 | 0.42 | 1.78 | 0.72 | | | | |
| | Non-infective enteropathy | 1.24 | 0.61 | 2.55 | 0.56 | | | | |
| | Inflammatory bowel disease | 1.65 | 0.49 | 5.54 | 0.42 | | | | |
| | Infective diarrhoea | 0.69 | 0.28 | 1.70 | 0.42 | | | | |
| Rheumatological involvement | Arthralgia | 1.24 | 0.57 | 2.71 | 0.59 | | | | |
| | Inflammatory arthritis | 1.81 | 0.73 | 4.46 | 0.20 | | | | |
| Cytopaenia | Any | 1.45 | 0.70 | 3.04 | 0.32 | | | | |
| | ITP | 1.54 | 0.72 | 3.27 | 0.26 | | | | |
| | AIHA | 1.57 | 0.47 | 5.25 | 0.46 | | | | |
| | AI neutropaenia | 0.60 | 0.08 | 4.43 | 0.61 | | | | |
| Thyroid disease | | 1.56 | 0.47 | 5.21 | 0.47 | | | | |
| Exposure to immunosuppression | Any | 1.87 | 0.90 | 3.90 | 0.10 | | | | |
| | Corticosteroids | 2.08 | 1.00 | 4.34 | 0.05 | 2.28 | 0.96 | 5.41 | 0.06 |
| | Mycophenolate mofetil | 1.94 | 0.58 | 6.48 | 0.28 | | | | |
| | Rituximab | 1.40 | 0.49 | 4.01 | 0.54 | | | | |
| | Azathioprine | 1.64 | 0.22 | 12.09 | 0.63 | | | | |
| | Ciclosporin | 1.52 | 0.20 | 11.44 | 0.69 | | | | |
| | Anti-TNF | 2.49 | 0.86 | 7.22 | 0.09 | 0.84 | 0.25 | 2.77 | 0.77 |
Supplementary table 8: Cox-proportional hazard models for the risk of death in the population of CVID patients excluding those diagnosed in the 1960s and 1970s
